# Supplementary material for: The feasibility, acceptability, and preliminary impact of real-time monitors and SMS on tuberculosis medication adherence in southwestern Uganda: Findings from a mixed methods pilot randomized controlled trial
Source: PLOS Glob Public Health. 2023 Dec 5;3(12):e0001813. doi: 10.1371/journal.pgph.0001813 (PMC10697590; doi:10.1371/journal.pgph.0001813)
Supplement: S2 Checklist — (DOCX) [file pgph.0001813.s002.docx]

**COREQ Checklist**

| **Domain 1:**   \| **Research team and reflexivity** \| \| --- \| |  | \| Location in manuscript (Section, page no.) \| \| --- \| |
| --- | --- | --- | --- | --- |
| **Personal characteristics** |  |  |
| 1. Interviewer/facilitator: Which author/s conducted the interview or focus group? | **WT, ATM** | “data collection” section, page 10 |
| 1. **Credentials:** What were the researcher’s credentials? E.g. PhD, MD | **PhD, MBA respectively** | Data Collection section, Page 10 |
| 1. What was their occupation at the time of the study? | **Research Assistants** | Data Collection section, Page 10 |
| 1. **Gender:** Was the researcher male or female? | **Male** | Data Collection section, Page 10 |
| 1. Experience and training: What experience or training did the researcher have? | At the time of the interviews, the researchers had completed training in:   1. Responsible conduct of Research 2. Qualitative research methods were covered at length 3. Research ethics by the Collaborative Institutional Training Initiative (CITI Program) 4. Data management | “data collection” section, page 10 |
| **Relationship with participants** |  |  |
| 1. Relationship established: Was a relationship established prior to study commencement? | Prior to data collection, the researchers had interacted with the participants at enrollment. | data collection” section, page 10 |
| 1. Participant knowledge of the   Interviewer: What did the participants know about the researcher? e.g. personal goals, reasons for doing the  research | Participants were informed about the purpose of the study. All participants were briefed on the purpose of the study and understood that it was a research project aimed investigating the use of: i) real-time adherence monitors, ii) SMS as adherence reminders to People living with TB, and iii) SMS notifications to their social supporters to support TB medication adherence in southwestern Uganda. They were informed at enrollment about their right to refuse/withdraw from the study at any time without any penalty or losing the benefits they were entitled to at the hospital facility.  They provided signed informed consent before study participation | Ethical approval- page12 |
| 1. Interviewer characteristics: What characteristics were reported about the interviewer/facilitator? e.g. Bias, assumptions, reasons and interests in the research topic | No interviewer-related biases identified | **-** |
| **Domain 2: study design** |  |  |
| Theoretical framework |  |  |
| 1. Methodological orientation and Theory: What methodological orientation was stated to underpin the study? e.g. grounded theory,   discourse analysis, ethnography, phenomenology, content analysis | Inductive content analytic approach | “Data analysis” section: Page 11 |
| **Participant selection** |  |  |
| 1. Sampling: How were participants selected? e.g. purposive, convenience, consecutive, snowball | We used purposive sampling to achieve relatively balanced representation by gender. | “Selection of study participants”: Page 7 |
| 1. Method of approach: How were participants approached? e.g. face-to-face, telephone, mail, email | Face-to-face | “data collection” section, page 10 |
| 1. Sample size: How many participants were in the study? | 102 | Results section, pages-12-13 |
| 1. Non-participation: How many people refused to participate or dropped out? Reasons? | -One participant requested to be disenrolled,  -One refused to use the device,  -One turned out to have negative TB results. | Result—CONSORT diagram; page12 |
| **Setting** |  |  |
| 1. Setting of data collection: Where was the data collected? e.g. home, clinic, workplace | Data was collected in a private space at a research office near the Mbarara regional referral hospital | “data collection” section, page 10 |
| 1. Presence of non-participants: Was anyone else present besides the participants and researchers? | No |  |
| 1. Description of sample: What are the important characteristics of the sample? e.g. demographic data, date | of 63 PLTB, 32 (59%) were male, 53 (84%) had co-infection with HIV, and 47 (75%) had no regular income. Of the 42 social supporters, 28 (66%) were female, 16 (38%) had co-infection with HIV, and 32 (22%) had no regular income. | Table 1 |
| **Data collection** |  |  |
| 1. Interview guide: Were questions, prompts, guides provided by the authors? Was it pilot tested? | Yes | “data collection” section, page 10 |
| 1. Repeat interviews: Were repeat interviews carried out? If yes, how many? | No |  |
| 1. Audio/visual recording: Did the research use audio or visual recording to collect the data? | Yes. Interviews were digitally recorded, transcribed, and translated to English | “data collection” section, page 10 |
| 1. Field notes: Were field notes made during and/or after the interview or focus group? | No additional field notes were made |  |
| 1. Duration: What was the duration of the interviews or focus group? | Each interview lasted between 30 and 60 minutes | “data collection” section, page 10 |
| 1. Data saturation: Was data saturation discussed? | Interviews were carried out until thematic saturation was reached at the 30^th^ participant and there was no new themes coming out from the data except repetition | “data collection” section, page 10 |
| 1. Transcripts returned: Were transcripts returned to participants for comment and/or correction? | No |  |
| **Domain 3: analysis and findings** |  |  |
| Data analysis |  |  |
| 1. Number of data coders: How many data coders coded the data? | One | “data analysis” section, page 10-11 |
| 1. Description of the coding tree: Did authors provide a description of the coding tree? | Inductive content analysis. (Theme identification, elaboration and illustration of quotes) | “data analysis” section, page 11 |
| 1. Derivation of themes: Were themes identified in advance or derived from the data? | Derived from the data | “data analysis” section, page 11 |
| 1. Software: What software, if applicable, was used to manage the data? | NVIVO | “data analysis” section, page 10 |
| 1. Participant checking: Did participants provide feedback on the findings? | No |  |
| Reporting |  |  |
| 1. Quotations presented: Were participant quotations presented to illustrate the themes / findings? Was each quotation identified? E.g. participant number | Yes, specific themes were supported with illustrative quotes attributed to anonymized participant details. | Table 4 |
| 1. Data and findings consistent: Was there consistency between the data presented and the findings? | Yes | “results” section, Table 4 |
| 1. Clarity of major themes: Were major themes clearly presented in the findings? | Yes | “results” section, page 19 |
| 1. Clarity of minor themes: Is there a description of diverse cases or discussion of minor themes? | Yes | “results” section, page 19 |
