# Supplementary material for: The feasibility, acceptability, and preliminary impact of real-time monitors and SMS on tuberculosis medication adherence in southwestern Uganda: Findings from a mixed methods pilot randomized controlled trial
Source: PLOS Glob Public Health. 2023 Dec 5;3(12):e0001813. doi: 10.1371/journal.pgph.0001813 (PMC10697590; doi:10.1371/journal.pgph.0001813)
Supplement: S1 Appendix — (DOCX) [file pgph.0001813.s003.docx]

Univariable and multivariate analysis of adherence across arms

|  | Univariable | | Multivariate | |
| --- | --- | --- | --- | --- |
|  |  | P | Co-efficient (CI) | P-value |
| Study arm |  | 0.50 |  | 0.41 |
| Control | Ref |  |  |  |
| Arm A | 8.4 (-5.8, 22.5) |  | 9.9 (-4.8, 24.5) |  |
| Arm B | 4.7 (-10.3, 19.8) |  | 6.3 (-8.7, 21.3) |  |
| Age | 0.01 (-0.01, 0.02) | 0.72 |  |  |
| Female | 7.5 (-3.6, 18.7) | 0.18 | 8.8 (-2.7, 20.2) | 0.13 |
| Education |  | 0.12* |  |  |
| None | Ref |  |  |  |
| P1-P7 | -7.7 (-20.6, 5.0) |  |  |  |
| >P7 | -10.4 (-22.0, 1.3) |  |  |  |
| Marital status |  |  |  |  |
| Coupled | Ref |  |  |  |
| Single | 3.1 (-8.0, 14.2) | 0.58 |  |  |
| Type of residence |  |  |  |  |
| Rural | -0.6 (-12.0, 10.9) | 0.92 |  |  |
| Town | Ref |  |  |  |
| Severe food insecurity | -8.9 (-20, 2.6) | 0.13 | -8.1 (-20.0, 3.9) | 0.18 |
| Disclosed status | -7.8 (-18.0, 2.4) | 0.13 | -8.3 (-18.8, 2.1) | 0.12 |
| Heavy alcohol consumption | -9.1 (-29.1, 10.8) | 0.36 |  |  |
| Low social support | -3.1 (-19.1, 12.8) | 0.70 |  |  |
| Stigma (disclosure concerns) | 1.76 (-1.2, 4.7) | 0.23 |  |  |
| Depression | -0.9 (-19.2, 17.5) | 0.93 |  |  |
| Asset index | 5.8 (-9.5, 21.2) | 0.45 |  |  |
| HIV co-infection | -9.4 (-20.6, 1.71) | 0.096 | -8.5 (-20.3, 3.4) | 0.16 |

*Unstable model so excluded from the multivariable variable
