# Supplementary material for: The feasibility, acceptability, and preliminary impact of real-time monitors and SMS on tuberculosis medication adherence in southwestern Uganda: Findings from a mixed methods pilot randomized controlled trial
Source: PLOS Glob Public Health. 2023 Dec 5;3(12):e0001813. doi: 10.1371/journal.pgph.0001813 (PMC10697590; doi:10.1371/journal.pgph.0001813)
Supplement: S1 File — (DOC) [file pgph.0001813.s004.doc]

| **FOR OFFICE USE ONLY** | Date of submission | Date considered | Approval granted? |
| --- | --- | --- | --- |
| Application No. ***(Yr/No)*** |  |  | yes / no |
| Signature:  (MUST-IRC Chair) |  |  |  |

THIS FORM MUST BE TYPEWRITTEN

# MBARARA UNIVERSITY OF SCIENCE AND TECHNOLOGY

**APPLICATION FORM FOR INSTITUTIONAL ETHICAL APPROVAL**

**ALL** QUESTIONS MUST BE ANSWERED. ANY FORM STATING "SEE PROTOCOL" WILL BE RETURNED. (This form must stand complete in itself).

PLEASE PROVIDE COPIES OF THIS FORM AND THE ORIGINAL PROPOSAL AS STATED IN THE GUIDELINE

AS FAR AS POSSIBLE YOU SHOULD RESTRICT ALL ENTRIES TO THE SPACE PROVIDED ON THIS FORM

Please use a typing font that is easily distinguishable from the questions of the form

NB This form is available on diskette from the MUST-IRC Office

| **NAME OF APPLICANT: Angella Musiimenta, PhD** |
| --- |

Have you submitted this proposal to the relevant Faculty/Institute Research Committee before? NA

Date and outcome: NA

If you are re-submitting a proposal, please emphasize how the proposal has been amended in the light of previous recommendations from the Faculty Research Committee or Institutional Ethical Review Committee. NA

If this proposal is for work that will go towards a higher degree (e.g. M.Med or PhD), please state name and Department of Supervisor(s): NA

**SECTION A**

**STUDY OUTLINE**

**A.1 TITLE OF PROJECT: Real-time Tuberculosis Medication Adherence Intervention in Rural Southwestern Uganda**

**A.2 SUMMARY**

Explain why this study is being conducted, using lay terminology.

*Guidance note:*

*Please convey what you think is the importance of the research and WHY it is being carried out.*

**A.2.1 Background**

With an estimated 60,000 people diagnosed with tuberculosis (TB) annually, Uganda holds the 16^th^ position of the 22 countries with highest cases of TB in the world [8]. The incidence of TB globally is 8.6 million with Uganda’s incidence cases estimated to be 65,000 and 4700 deaths [14]. High TB prevalence in Uganda can result into high TB infections rates, high burden on the already struggling healthcare systems, and negative impacts economic development since a sickly population cannot contribute to the country’s economic development. The importance of high TB medication adherence for good clinical outcomes is well known [8,9,15]. TB treatment is widely available in Uganda, however, significant adherence challenges remain, thus constraining TB treatment success for the individual and increasing its transmission [16]. Treatment non-adherence puts patients at a high risk of developing drug-resistant strains of TB, which are more expensive to treat and difficult for patients to comply with, given the longer duration of treatment and many side effects. Uganda currently has 400 multi-drug resistant TB patients [14]. It costs about $4,000 to treat a multi-drug resistance TB patient for a period of 24 months versus $80 to treat a drug susceptible patient for six months. Interventions to prevent drug resistance are urgently needed. Causes of non-adherence to TB medication include lack of patient follow-up [8], patients’ lack of transport to go to the clinics to pick up drugs, patients’ forgetfulness [10], and inability to implement DOTS strategy due to limited resource [9].

The Uganda national target of utilizing the DOTS (Direct Observed Treatment Short Course) to successfully treat 85% of patients diagnosed with TB has not been met. Currently, the country only detects approximately 50% of TB cases, of which it successfully treats 73% [8]. The DOTS strategy has suffered challenges of shortage of human and economic resources, which have resulted into its abandonment by many of the Ugandan hospitals [9].

In Uganda, over 70% of people have personal mobile phones [19]. The rapidly expanding cellular networks and wider adoption of cell phones including rural areas in Uganda has greatly increased the potential of cellular technology to serve as a novel solution to challenges of medication adherence. Technology can play an important role in addressing barriers to adoption of good health behaviors [3]. Our recent study that explored the use of real time adherence monitoring linked with SMS reminders and social support notifications demonstrate the potential benefit of such integrated interventions in addressing barriers to sustained ART adherence [45]. This type of intervention could potentially also work for adherence to TB medication, and such novel interventions addressing TB medication adherence challenges in low resource settings to date are limited [10]. The prevailing SMS-based studies for TB medication adherence report mixed results, do not strategically link interventions with missed doses, and have largely been implemented in developed countries [11-12 ] To date, little is known about the use of real-time adherence monitoring technologies for TB medication adherence in resource-limited settings.

Real-time adherence monitoring creates the potential for intervening prior to the development of treatment failure and/or drug resistance. Traditional approaches to adherence monitoring, such as self-report and clinic-based pill counts, identify adherence challenges weeks to months after they occur, which may result in drug resistance [10]. Real-time monitoring with wireless devices enables the delivery of targeted interventions when and where they are needed. It has the potential to change treatment monitoring from a reactive response to a proactive prevention of TB treatment failure and/or drug resistance. This approach has profound impact in Sub-Saharan Africa where second regimens for drug resistance are scarcely available and largely unaffordable [8]. However, few studies have explored it to date.

The approach of using targeted intervention based on real-time monitoring of the individual’s adherence behavior is consistent with a paradigm in healthcare delivery called “just-in-time”, which is receiving increasing attention. It refers to the provision of services in the moment they are needed. Recently, our group has shown this approach to be effective in individuals initiating HIV ART adherence [45], and has great potential to be applied to TB medication adherence research. Although a lot can be learnt from this experience, HIV requires lifelong therapy, and the differences in the stigma associated with HIV and TB many imply differences in adherence strategies required. To my knowledge, this study is the first in sub-Saharan Africa to combine SMS reminders, social networks to provide individualized support, and real-time adherence monitor to support TB medication adherence. Social networks may be the critical means of community functioning, providing support (e.g. affective, monetary, informational) to TB patients in the absence of developed health systems, and this has particular significance in Uganda, low-resource country. Using technology to precisely match adherence support with missed dozes and leveraging the social support may be an important resource for overcoming barriers to adherence.

This study is innovative in its concurrent pairing of intervention development with qualitative analysis to understand the mechanisms by which SMS reminders and automated engagement of social networks may influence adherence behavior. The mechanism (e.g. content, timing, frequency) by which SMS reminders influence TB medication adherence remains unclear. This knowledge will make it possible to design and test evidence-based TB medication interventions with the highest likelihood for efficacy. The positive impacts of such intervention on ART adherence are reported [45-47], although there has also been mixed results as one in China showed an improvement in overall ART adherence [20], whereas one in South Africa did not [21]. Understanding the application of such novel interventions in TB medication adherence is crucial since TB is the leading killer among HIV/AIDS patients [9].

**A.3 OBJECTIVES/SPECIFIC AIMS**

List the major objectives/hypothesis, which have governed your choice of study design

**Aim 1: Carry out a formative qualitative study to assess barriers and facilitators to TB medication adherence, and identify optimal SMS reminders, notifications, and initial feasibility of real time adherence monitoring**. I will recruit up to 25 TB patients recruited from the TB clinic in Mbarara Regional Referral Hospital (MRRH), and up to 25 social supporters specified by the TB patients (one per TB patient).Drawing from technology adoption and behavioral change models [7, 48] , we will use semi-structured interviews to assess the initial feasibility and acceptability of the intervention, as well the barriers and facilitators to TB medication adherence, and how they can be addressed using the proposed intervention. The purpose will be to inform the development the technology necessary to conduct a real-time intervention involving a wireless monitor and SMS reminders and notifications (*Aim 2 and 3*).

**Aim 2: Develop a TB medication adherence intervention based on SMS reminders (fixed and/or linked to real-time detection of missed doses) and assess its acceptability, feasibility and preliminary impact on adherence.**

We will use real-time electronic adherence monitoring (i.e., Wisepill) to follow 60 TB-infected individuals initiating TB treatment in Mbarara Regional Referral Hospital (MRRH) for six months.

We will randomize these participants in a 1:1:1 manner to one of the following three study arms (details of each intervention will be informed by the data collected in Aim 1):

- 1. Intervention Arm A: SMS reminders on a fixed schedule
  2. Intervention Arm B: SMS reminders linked to missed doses
  3. Control: No SMS reminders.

We will conduct qualitative interviews to understand their experiences with Wisepill adherence monitoring and the SMS reminders, depending on the study arm to which they are assigned. We plan to conduct the first set of interviews for Arms A and B at the end of Month 2 of the study. The second set of interviews is planned to be conducted within two weeks after the first 48+ hour lapse in Wisepill signal for the participants in the intervention arms (A and B). If there is no 48+ hour lapse, the interview will take place at the end of the study. The control participants in Arm C’s interview will occur at a convenient time during Months 3-6. We will also estimate effect sizes by comparing adherence at the end of six months between each intervention group and the control group. The primary outcome will be percent adherence. Treatment outcome will be assessed as secondary outcomes. Treatment outcomes will be defined according to WHO definitions, where cure and completed treatment are defined as successful treatment outcomes (WHO 2014). Unsuccessful treatment outcomes for active TB treatment will include death ascertained from hospital death registry, treatment failure (sputum smear positive at 6 months) and loss to follow-up. Treatment outcomes will be compared for both intervention and control arms.

***Aim 3:* Develop a social support intervention linked to real-time adherence monitoring and assess its acceptability, feasibility and preliminary impact on adherence.** We will recruit members of pre-existing social support networks for the same 40 TB-infected individuals in the two intervention arms described in Aim 2 above to receive real-time SMS notification of sustained non-adherence (i.e., gaps of >48 hours) over the latter four months of follow-up. We will continue to follow the 20 TB-infected individuals in the control group (i.e., electronic monitoring alone) to estimate the effect size of the intervention on adherence, as well as viral suppression, at the end of the six-month period. We will conduct qualitative interviews to understand the experiences of participants in each study arm and the social supporters with special emphasis on understanding mechanisms of effects of the SMS reminders, forms and dynamics of support, experiences with the SMS reminders/notifications, and technical problems encountered. We will use the results of Aim 3 to determine the types of SMS (fixed versus linked SMS), and the nature of social support that show the most promise for real time intervention on anti-TB medication adherence and cure, which will fully be evaluated through an R01 supported randomized control trial.

Fig 1. Intervention overview: All participants in the 3 arms will have Wisepill adherence monitoring

Interviews at end of Month 2

Interviews between month 3 and 6

| Intervention Arm A (N=20) | Daily SMS Weekly SMS | SMS for missed dose + Social support notifications  (4 months) |
| --- | --- | --- |
| Intervention Arm B (N=20) | SMS with missed dose | SMS for missed dose + Social support notifications  (4 months) |
| Control Arm (N=20) | No SMS  Interviews between month 3 and 6 | |

**A.4 METHODOLOGY**

Outline how you intend to achieve the objectives of the study.

*Guidance notes:*

*For each objective/hypothesis:-*

- *define the target population*
- *describe how the sample(s) is(are) to be recruited from the target population(s)*

*Even if the main thrust of the research is biomedical, the rationale behind your use of social science methods (e.g. patient interviews) should be clear.*

*Aim 1*

Target populations: Up to 25 TB patients undertaking treatment at MRRH, and up 25 social supporters (as identified by the TB patients, one per TB patient).Recruitment of samples: Purposeful sampling based on the criteria outlined in A.5.3

*Aim 2*

Target population: 60 newly diagnosed TB patients initiating treatment at MRRH, which will include 20 participants in Arm A, 20 in Arm B, and 20 in the Control Arm.

Recruitment of samples: Purposeful sampling based on the criteria outlined in A.5.3

*Aim 3*

Target population: The same 40 newly diagnosed TB patients initiating treatment at MRRH in Arms A and B of the interventions described in Aim 2, and their 40 social supporters (one per Wisepill participant—Wisepill participants are TB patients who will be given the wisepill).

Recruitment of samples: Purposeful sampling based on the criteria outlined in A.5.3

**A.5 PARTICIPANTS**

Please provide the following information on the participants with/from whom you expect to be collecting data:

A.5.1 Age / Sex: (please enter the expected number in each of the boxes)

|  | Neonates  (<28 days) | Infants  (1-11 months) | Young children  (1-9 years) | Adolescents  (10-17 years) | Adults  (18 yrs & above) |
| --- | --- | --- | --- | --- | --- |
| **Males** |  |  |  |  | up to 70 |
| **Females** |  |  |  |  | up to 80 |

*Guidance notes:-*

*This age/sex breakdown helps convey how vulnerable the participants will be*

*If you are unable to give precise figures, state estimates and give an explanatory sentence in the space below*

We will use purposeful sampling (prior to randomization) to reflect both genders and ages less than and greater than 30 years, which may have different views and experience with cellular technology.

A.5.2 What specific measures are in place to take into account women of childbearing age?

*Guidance notes:*

*Pregnant women may have different responses to disease processes*

*The developing foetus may be particularly vulnerable in intervention trials*

Women of childbearing age will be included as long as they meet the inclusion criteria outlined in A.4 above.

A.5.3 Describe how and where the participants are to be recruited?

A.5.3 *Guidance notes:*

*This is distinct from the statistical sampling method described in A.4. You should outline the procedures for recruitment of each group of participants, include details on:*

- *the setting (e.g. Country, Town, District, on the ward, out-patient department ,in the home)*
- *inclusion and exclusion criteria for selection, if relevant (e.g. “Women of child-bearing age will be excluded”)*

The study will be conducted at TB clinic in Mbarara Regional Referral Hospital (MRRH) in Mbarara, Uganda. The TB clinic provides care to about 600 TB patients. All newly diagnosed patients receive standard care of free and accessible TB medication, and counseling on the benefits of TB medication. Apart from patients with the drug resistant TB, all patients self-administer their medications—they are not on DOTS strategy. The clinic policy is to give newly diagnosed TB patients pills every after two weeks during the first two months of treatment. They then return to the clinic in Month 2 for conversion checking. After Month two, they pick pills every month up to six months. At four months, they come to the clinic for conversion checking, and then at six months for final check-up.

We will use the following criteria for identifying participants:

Inclusion criteria for *Aim 1*

*Wisepill participant*

- Infected with TB per the TB Clinic records
- TB patient infected with HIV/AIDS will also be included.
- Own a cell phone
- Been undergoing a first-line 6 months course of anti-TB therapy (standard TB care of treatment and counseling) for at least a month.
- Age 18 years and older
- Lives in the Mbarara District (20 km from MRRH)
- Willing and able to give consent
- Willing and able to name one social supporter

*Social Supporters*

- Know a Wisepill participant and be aware s/he has TB
- Have provided help to that Wisepill participant at least once. By “help”, we mean things like
- Helping him/her get to clinic by loaning or giving money, driving the patient , or taking care of his/her job or children while he/she is away
- Helping the patient take medicines through encouragement or reminders
- Motivating the patient to take medicines, including addressing cognitive and behavioral barriers such as depression and alcohol use
- Age 18 years or older
- Live in Mbarara District (within 20 KM from MRRH)
- Willing and able to provide consent.

Exclusion:

- Unable to use SMS (we will train and test this skill at recruitment)
- Unwilling to receive SMS reminder
- Severe mental condition limiting the ability to provide consent
- Cellular phone reception is not reliable

Inclusion criteria for *Aim* 2 *and Aim 3*

Wisepill participants:

- Newly diagnosed with TB per the clinic records
- Starting TB treatment now or within the next two weeks
- Age 18 years and older
- Live in the Mbarara District (20 km from MRRH)
- Own a cell phone for personal use and have reliable cellular phone reception at home on networks (MTN or Airtel) supported by the technology used in this study
- Know how to use SMS
- Willing and able to give consent
- Willing and able to name one or two social supporters who are able to use SMS and have cellular phones using network provider (MTN or Airtel) supported by the technology used in this study

Exclusion:

- Unable to use SMS (we will train and test this skill at recruitment)
- Unwilling to receive SMS reminder
- Severe mental condition limiting the ability to provide consent
- Cellular phone reception is not reliable

Because approximately 70% of patients in the TB clinic own cell phones and live within Mbarara District, we do not believe these enrollment criteria will unduly bias or limit the generalizability of the study findings.

Social Supporters:

We will use the same criteria as the one used for social supporters of *Aim 1* above.

The social supporters may be friends, family or otherwise, as long as they meet these criteria. We will carefully review the study and expectations for participation with both the Wisepill participant and the social supporter to make sure enrollment is appropriate.

A.5.4 Please justify your choice of sample size (as described in A.4)

The proposed 150 participants are enough to provide the required initial feasibility, acceptability and impact of the intervention. For qualitative aspects, we plan to continue to enroll participants until interviews do not reveal additional themes.

For *Aim 1*, based on prior studies [45-47], we estimate that we will achieve thematic saturation with the proposed 50 participants. Additionally, we plan to continue to enroll participants (up to 50) until interviews do not reveal additional themes.

*Aim 2 and 3*: The goal of this aim is to demonstrate initial acceptability, feasibility and preliminary impact. In our prior work with this type of intervention, we were able to meet these goals with 60 Wisepill participants and 40 social supporters [45-47].

**A.6 PROCEDURES**

A.6.1 What procedures or methods will be employed in the collection of data (e.g. patient interviews / focus group discussions / blood sampling / biopsies) and by whom (e.g. experienced facilitator / social scientist / teacher/ qualified doctor / nurse, auxiliary, etc.)?

Attach additional sheets if necessary.

| **Procedure** | **To be carried out by:** |
| --- | --- |
| Conducting interviews and their transcription | Experienced PI and RA |
| Review of interview transcripts | Experienced PI and Mentor (Jessica Haberer) |
| Survey administration | Experienced PI and RA |
| Assessment of TB cure | Experienced Mentor (Luke Davis, Cornrad Muzoora) |
|  |  |

*Overview of Data Collection*

*Aim 1: Interviews for formative research*. An single in depth interview will be carried out with up to 25 TB patients, and up to 25 social supporters assess the initial feasibility and acceptability of the intervention, as well the barriers and facilitators to TB medication adherence, and how they can be addressed using the proposed intervention. The participants for *Aim 1* will only be interviewed once, and they will not be the same participants used in *Aims* 2 & 3. Additionally, a short survey capturing sociodemographic details, telephone use, and TB/HIV status will be administered to the formative research participants. Enclosed are the interview guide, and the survey instrument.

*Aims 2 and 3: Interviews for Wisepill participants*. Two in depth qualitative interviews will be conducted per Wisepill participant to understand their experiences with Wisepill adherence monitoring, the SMS reminders, and/or the social support intervention, depending on the study arm to which they are assigned. We plan to conduct the first set of interviews for Arms A and B at the end of Month 2 of the study. The second set of interviews is planned to be conducted within two weeks after the first 48+ hour lapse in Wisepill signal for the participants in the intervention arms (A and B), between 3 and 6 months. If there is no 48+ hour lapse, the interview will take place at the end of the study. Participants in the control Arm C will be interviewed once, and this interview will occur at a convenient time during Months 3-6. Some variation in timing may occur due to logistical considerations and the transition to the second type of intervention may be delayed accordingly. The interviews will take place either at the participant’s home or the study offices, per the participant’s preference, and will last approximately one hour or less. The interviews will be digitally recorded for transcription, after which time the recording will be deleted. Enclosed is the content of the interview guides.

The second interview will be conducted within two weeks after the first 48+ hour lapse in Wisepill signal for the intervention arms (A and B) between month 3 and 6.

If there is no 48+ hour lapse, the interview will take place at the end of the study. The control participants in Arm C’s interview will occur at a convenient time during Months 3-6.

We will also administer a brief exit interview to participants in the intervention arms (A and B) at disenrollment to learn about the overall experience with the intervention. This interview may take place over the phone, at the participant’s home, or at the research offices, per the participant’s preference.

*Interviews for social supporters.* Up to 40 social supporters (will be given in depth qualitative interviews within two weeks after the first 48+ hour lapse in Wisepill signal for the Wisepill participant with whom they are associated. These interviews will take place either at the participant’s home or the study offices, per the participant’s preference, and will last approximately one hour or less. The interviews will be digitally recorded for transcription, after which time the recording will be deleted. See the Appendix for the content of the interview guide.

All social supporters will be interviewed briefly at the end of the study to obtain a brief description of the assistance provided, if any. This data will be used to categorize the assistance as emotional, instrumental, material, or other. This interview may take place over the phone, at the participant’s home, or at the research offices, per the participant’s preference.

Table 1: Number of Participants

| **Aim** | **Number of participants** |
| --- | --- |
| 1 | 25 patients and 25 social supporters |
| 2 and 3 | 60 patients and 40 social supporters for |
| **Total Participants** | **150** |

*Overview of other study procedures*

a. *Randomization.* Once eligibility has been confirmed for the Wisepill participants, we will randomize each participant to one of the three arms of the study (two intervention arms and one control as described above). To do so, we will randomly order the numbers 1-60 via a random number generator (www.randomizer.org) prior to initiation of the study and divide them into three consecutive groups of 20, each of which will be labeled as Intervention Arm A, Intervention Arm B, or control.

These number sets will be kept by the study PI and used after the completion of enrollment. Participants will then be informed as to which study arm they will be in via sealed envelope. Sequential randomization will be verified through periodic checks by a co-investigator.

b. *Enrollment of Wisepill participants*. We will enroll participants within two weeks of completing consent; participants not enrolled within this time period will be asked to repeat the consenting process if they still wish to participate. Baseline procedures will include training on the Wisepill device for adherence monitoring, as well as completing the questionnaire packet.

Training on the Wisepill device will include the following key points:

- The device will automatically send a cellular signal to the Internet every time it is opened. The lights on the side of the device will blink during this process.
- The device will automatically send a signal around midnight to indicate it is working, even though it has not been opened.
- Participants should only store TB medication in the device.
- Participants should open the device only when taking a dose of antiretroviral medication.
- Participants should transfer medications (e.g., refills) at the time a dose is taken.
- The device should not be submerged in water; wiping it off with a damp cloth is ok.
- Participants should flash (i.e., call and hang up before incurring any costs) their research assistant if any technical problems are suspected (e.g., the lights not blinking with each opening)
- Participants should charge the Wisepill battery every month by electrical outlet. The research assistant may contact them if the battery is getting low and assist with charging or changing the battery.
- The Wisepill pill container must be returned to the study at disenrollment.

Questionnaire packet CRFs (see Appendix): The following questionnaires will be administered before interview one to Wisepill participants in Arm A and B. Technology adoption will also be administered at 2 months.

At enrollment, social supporters will also complete all the following questionnaires with exception of technology acceptance questionnaire.

The questionnaires are:

- Demographics, cellphone use, and health (including HIV and TB status)
- Socio-economic status
- Food security
- Depression
- Alcohol use
- Social support
- Stigma
- Technology acceptance

1. *Wireless adherence monitoring with Wisepill.* The Wisepill system will automatically capture and report each time the device is opened as a proxy for the participant’s adherence. Data is sent by GPRS (general packet radio service, as is standard for most data transmission) with SMS back up in the case of poor cellular network availability. Data is also stored on the device using flash memory in the event cellular network is not available and will be automatically sent once adequate cellular network is available. We will monitor for technical functionality: e.g. Devices without a heartbeat for 3 days will be flagged. Persistent lack of a heartbeat for 7+ days will trigger a phone call or home visit to assess function. Data will be stored on a secure server with password-protected web-based access.

e. *Fixed SMS reminders.* SMS reminders will be sent on a schedule (e.g., daily, weekly per the formative data collected in Aim 1) to Wisepill participants in the Intervention Arm A automatically through the Dimagi SMS platform called CommConnect via an SMS gateway at Yo! Voice Solutions in Kampala Participants will indicate the content they would like to receive for the SMS reminders at enrollment and will be given the opportunity to modify their content at any time during the study upon request. Data will be stored on a secure server with password-protected web-based access.

f. *Triggered SMS reminders.* Triggered SMS will be through the Dimagi SMS platform called CommConnect via an SMS gateway at Yo! Voice Solutions in Kampala. They will be sent to Wisepill participants in Intervention Arm A starting at Month 3 of the intervention study and continuing until month 6 of the intervention study. And for participants in Intervention Arm B throughout the study, SMS reminders will be automatically sent from the Wisepill system when no signal is received during a dosing window (e.g., one hour before or after an expected time).

g. *Enrollment of social supporters.* We will enroll these participants within two weeks of completing consent; participants not enrolling within this time period will be asked to repeat the consenting process if they still wish to participate. We will first provide background information on the Wisepill and SMS reminder systems. We will then explain the social support intervention (see below). The procedures and specific guidance/support to be given to social supporters in preparation for their roles in this study will be based on the findings from the formative investigations in Aim 1.

Additionally, the following questionnaire packet CRFs will be completed (at enrolment) before the interview (see Appendix):

- Demographics/cellphone use/relationship to the Wisepill participant
- Socio-economic status
- Food security
- Depression
- Alcohol use
- Social support
- Stigma

h. *SMS notification for social supporters.* Starting at Month 3 of the intervention study, the Wisepill system will automatically generate reports if no openings are received for TB-infected individuals in the intervention arms (A and B) for 48+ hours or within a week. These reports will be used to send SMS notifications to their social supporters, asking them to provide assistance, if possible. We will not give specific instructions because we wish to observe how the participants chose to provide support (e.g., emotional, instrumental, and/or material assistance), but they will be advised to treat the SMS notification like a request for help. An SMS notification will be sent at the beginning of the 48+ hour lapse in signal and repeated one time if the lapse is still continuing one week later.

*Medical record review*: Medical records will be reviewed to get information about participants’ conversion, gaps in adherence, TB cure rate, treatment completion rate, clinic appointment attendance, and mortality.

*Exit Interviews*: We will administer quantitative exit surveys with the wisepill participants and social supporters to understand their experiences with the intervention.

A.6.2 State the extent to which the procedures to be used are a part of usual clinical management (if appropriate).

Procedures will not be used as part of clinical management.

A.6.3 Please indicate that the persons identified in A.6.1 are competent to carry out these procedures. List any training of staff that may be required prior to commencement of the study.

All are competent in data collection and research ethics. Where need be, specific training in qualitative interview techniques will be provided.

**A.7 ANALYSIS**

A.7.1 What are the major statistical (or other) methods that you intend to use to analyse the data to

fulfill each of the objectives/hypothesis stated in A.3

1. *Quantitative data*

*Study population characteristics*. We will use summary statistics to analyze the health-related and sociodemographic data described above. Although the populations should be similar due to the randomization, we will assess for underlying differences that may affect our conclusions using T-tests (or Mann-Whitney U tests, depending on the normality of the data) for continuous data and chi-squared tests for categorical data.

*Technical feasibility of the intervention*. We will verify the technical function of the intervention with the following statistics: Number of SMS received by the participant / number SMS anticipated per protocol, number and type of technical problems encountered, number of SMS notifications responded too by the social supporters.

*Adherence and effect size*. The primary outcome will be percent adherence. We will use SPSS or STATA, to determine the mean (standard deviation), median (interquartile range), number of late doses, number of missed doses, and number of 48+ hour gaps in adherence as determined by Wisepill for each stage of the intervention, as well as for the control group. We will compare adherence during each type of SMS reminder with adherence during the matched time period for the control group using T-tests (or Mann-Whitney U tests, depending on the normality of the data) for continuous data and chi-squared tests for categorical data. We will use the effect sizes to determine the sample size needed to show statistical significance within the planned, subsequent grant application.

*Social support adherence partners*. We will summarize social support demographic data and estimated *P*-values with chi-squared tests using a level of significance of 0.05. Poisson regression will be done to assess associations of categorical variables with patients missed dozes. Social supporter characteristics (i.e., type of relationship, income status, alcohol use, gender, involvement in community support group and TB status) will be selected as predictors of adherence based on potential impact on the relationship and on social support for the study participant.

*Secondary Analysis*: As a secondary analysis, we will assess gaps in adherence, cure rate, treatment completion rate, treatment success rate, clinic appointment attendance, using descriptive statistics. We will also determine the mortality from hospital’s death registry.

*Technology Acceptance*: We will use modeling-tests to explore the relationships between the various constructs of the technology acceptance model, and assess their effect on the acceptance of the intervention technology in this study.

1. *Qualitative data.*
2. We will use an inductive, content analytic approach for analysis of the qualitative data. Content analysis refers to a systematic process for interpreting the content of textual data through coding and category construction [23].
3. We will develop a coding scheme through initial review of a randomly selected subset of 33% of interview transcripts. Sections of text that appear to address concepts of analytic interest (e.g., acceptance, experience of SMS reminders) will be assigned descriptive labels, or codes. Operational definitions will be developed for the codes to create a coding scheme, which will be used to code the data. We will use the qualitative data management computer program (NVIVO) to code the data, which will then be repeatedly sorted and re-reviewed to identify a broader set of concepts (e.g., representing “acceptance” and “experience”). The categories will be constructed from this second, more general set of concepts through assignment of descriptive labels, formulation of operational definitions, and selection of illustrative citations from the data. Once the categories are in place, they will form the basis for a comparative analysis across phases and types of the intervention, to identify similarities and differences in interviewee responses. We will review categories and posit semantic links (e.g. causal links, chronological links) among them to characterize mechanisms of effect in the final, interpretive step of the analysis.

**A.8 QUALITY ASSURANCE**

A.8.1 What procedures are in place to ensure the quality of the data?

*Guidance notes:*

*For qualitative data (for example) what procedures will be used to check translations or compare data obtained from different sources?*

*For quantitative data (for example) how will transcription errors be minimised?*

*Give some detail on how methods are going to be piloted, if appropriate*

The PI will be responsible for continuous data and safety monitoring of all study participants. Serious adverse events (e.g. physical or emotional injury) will be reported to all involved IRBs according to the guidelines of MUST REC (i.e. within 14 days).

The PI will create a tracking spreadsheet to document enrolment, participation in the interview, completed questionnaires and other study-related documentation. It will be documented daily to enable timely review by the mentors. A representative sample of interviews transcribed by the Research Assistant will be re-transcribed by the PI to gauge fidelity in transcription and translation.

Quantitative data will be cleaned and validated before analysis

**A.9 DISSEMINATION OF RESULTS**

Please outline what plans you have for dissemination of results.

*Guidance notes:*

*Where possible a mechanism should be in place to inform study participants of the outcomes of the study.*

*It is important that study findings are made known to local services / policy makers before they are discussed (e.g.) at international scientific meetings*

We anticipate presenting the results of this study in international health informatics and TB conferences, and disseminating results via peer reviewed publications. Additionally, we plan to present the findings to study participants to inform them about the results of the study.

**SECTION B**

**CONSEQUENCES FOR THE LOCAL COMMUNITY / ENVIRONMENT AND PARTICIPANTS**

B.1 Outline the potential adverse effects, discomfort or risks that may result from the study in the following areas:

B.1.1 Participants

*Guidance note:*

*In addition to the physical effects of tissue sampling (for example blood sampling) it should be borne in mind that interviews and focus group discussions may sometimes trigger painful or distressing memories (e.g. questions about sexual practice or the death of a child)*

1. Loss of privacy and/or confidentiality. The most significant risk to participants is the potential for loss of privacy and confidentiality. We recognize the vulnerability of TB-infected individuals and their social supporters, and will take every possible precaution to protect their privacy and confidentiality. Use of the Wisepill adherence monitors, SMS, and home visits (i.e., at enrollment and for interviews, if desired) may involve some risk to the confidentiality of participants’ TB status in particular.

*2. Fatigue and/or anxiety.* Answering questions may cause participants to feel tired and/or some anxiety.

*3. Discomfort with adherence monitoring.* Some participants may feel uncomfortable with the idea of researchers monitoring their adherence electronically. Some participants may also feel concerned about others seeing them use the monitors.

*4. Relationship discord*. Participating in this study may affect the relationship between the Wisepill participants and social supporters depending on the support provided or not provided.

B.1.2 Investigators

*Guidance notes:*

*Include here (for example)*

- *the biomedical risks to investigators (including local staff) involved in tissue sampling (e.g. Hepatitis B, HIV)*
- *the psychological consequences for social science investigators exposed to narratives of violence or severe grief*
- *the risks from the environment (e.g. in a war zone)*

We do not anticipate risks to researchers during this study.

B.1.3 Members of the public

We do not anticipate risks to members of the public during this study.

**B.2 Outline what steps will be taken to minimize the adverse effects, discomfort or risks described above.**

B.2.1 For participants

*Guidance notes:*

*In biomedical research, appropriate use of anesthesia prior to procedures (for example) is important.*

*For social science research it may be necessary to ensure that counseling services are available for those who re-live traumatic experiences through (for example) an in depth interview.*

1. To minimize the risk of potential loss of privacy and confidentiality:
   - Participants may decline a home visit at any time if there are visitors or family members present who the participant does not want to witness the research procedures.
   - Research staff will go to the participants’ homes at a preferred, convenient time in an unmarked vehicle. We will maintain anonymity and safeguard participants confidentiality at all times.
   - All participant data entered via the computer or on CRFs will be associated with a participant identification number only.
   - A master list of participant names and their identification numbers will be stored in a locked file cabinet in the office of the Principal Investigator. The following additional steps will be taken to protect privacy:
   - All interviews will be conducted in private locations (i.e., at the participant’s home, study office or in another location of their choosing) outside of the TB clinic and without the presence of clinic staff.
   - The research assistant will receive specific training in procedures for protecting confidentiality from the study investigator.
   - The list of participant names and corresponding identification numbers will be stored in a location separate from the data.
   - All data stored electronically will be secured and protected by password.
   - All risks will be reviewed with participants during the informed consent process and strategies will be discussed as needed (e.g., where to confidentially store the Wisepill device, what to do when traveling with the device).

1. To address feelings of fatigue and/or some anxiety during answering questions:
   - Participants will be invited to take a break if they become tired.
   - If participants demonstrate apprehension or distress, they will be reminded of their rights to decline to answer any questions and to terminate the interview or their participation in the study at their discretion without any consequences (e.g., medical care at MRRH).
   - Participants who express distress will be referred to counselling services. Additionally, participants will be informed that the research staff will never discuss any identifiable information collected through this study with anyone outside of the study.
2. To address potential concerns with adherence monitoring:

- We will assure the participants that they will not be judged for lapses in the Wisepill signal, which could reflect technical challenges and/or adherence behavior.
- During consent, participants will be informed of the possibility of other people seeing the monitor.
- Participants will also be advised at consent and as needed during the study that they may disenroll from the study at any time without consequences.

1. To minimize relationship discord:

- Information will also not be shared between the TB-infected individuals and their social supporters.
- Participants who express distress will be referred to counselling services

Additional protections

- - As part of the introduction to each participant interview, we will make it clear that we are not visiting to provide medical care, nor do we have the expertise to answer health-related questions. All questions about the research will be completely and carefully answered; however, questions about medical care will be referred to health care providers.
  - If, during home visits, we find participants having medical problems, we will offer to provide transportation to the clinic.
  - Before each study activity participants will be reminded that they have the right to decline.

B.2.2 For investigators

*Guidance notes:*

*Where the research may involve adverse experiences for investigators (see B.3.2), de-briefing / support meetings may be important. NA*

B.2.3 For members of the public: NA

**B.3 CONSEQUENCES FOR LOCAL HEALTH SERVICES**

B.3.1 What demands will this research place on local health services?

*Guidance notes:*

*For example, how much of a nurse’s usual work time will be taken up in acting as an interpreter for an outside investigator?*

We do not anticipate additional demands to local health services during this study.

B.3.2 Detail how the design of the research project takes into account the demands described in 3.1.

*Guidance notes:*

*Disruption to routine services should be kept to a minimum.*

The secondary outcomes of TB cure rate, treatment completion rate, treatment success rate, clinic appointment attendance will be obtained from the clinic records with minimum interruptions (if any) since they will also be obtained on an appointment basis with healthcare providers.

**B.4 CONFIDENTIALITY AND PRIVACY**

B.4.1 What steps will be taken to ensure privacy and confidentiality for participants?

See section B.2 above

B**.5 INFORMED CONSENT**

B.5.1 Information given to participants:

Please indicate what you will tell the participants in simple language. The purpose of the study, type of questions that will be asked, and procedure or treatment which will be applied should be described and reference should be made to possible side effects, discomfort, complications and/or benefits. Please attach consent form typed on MUST-IRC official consent form.

| **It must be made clear to the participant that he/she is free to decline to participate or to withdraw at any time without suffering any disadvantage or prejudice.** |
| --- |

Participants will be informed that they may voluntarily withdraw from the proposed study for any reason at any time without affecting their health services. The researchers also may withdraw participants in order to protect their safety and/or if they are unwilling or unable to comply with required study procedures. Reasons for withdrawal will be recorded.

*Formative research patients*

TB patients will be identified with the assistance of healthcare professionals working in the TB clinic at MRRH. We will approach each of the identified patient to accompany him/her to a private space at the study office on the Mbarara University campus. Using the consent form and the language preferred by the patient, we will introduce the study and gauge the patient’s interest. Eligibility criteria will be reviewed per the participant screening case report form (CRF). Potential participants will be told the purpose of the study and the reasons they have been approached for participation. The study procedures will be explained, including the randomization process, as well as anticipated risks and benefits.

*Formative research social supporters*: The TB patients of the formative research will be asked to identify potential social supporters at enrollment. We will then contact these individuals by cell phone during the formative research of this study to see if they are potentially interested in participating. If so, we will make arrangements for them coming to the study offices for screening and, if appropriate, consent. All activities will be conducted in a private space. Using the consent form and the language preferred by the patient, we will introduce the study and gauge the individual’s interest. Eligibility criteria will be reviewed per the social supporter screening CRF. Potential participants will be told the purpose of the study and the reasons they have been approached for participation. The study procedures will be explained, including the anticipated risks and benefits. If an individual agrees to participate, we will complete the consenting process by asking the participant to sign the consent form. A copy will be provided to the participant. Transport will be refunded in cases where the potential participants come purposely for the consenting process.

*Wisepill participants.* Physicians, nurses, and/or pharmacists at the TB clinic in MRRH will identify eligible patients during routine clinic visits, as has been done for several years with a similar study with our previous studies. We will approach each of the identified patient to accompany him/her to a private space at the study office on the Mbarara University campus. Using the consent form and the language preferred by the patient, we will introduce the study and gauge the patient’s interest. Eligibility criteria will be reviewed per the participant screening case report form (CRF). Potential participants will be told the purpose of the study and the reasons they have been approached for participation. The study procedures will be explained, including the randomization process, as well as anticipated risks and benefits. We will emphasize the following: 1) all participants will receive electronic adherence monitoring devices; 2) some participants will be randomly assigned to receive SMS reminders for themselves; 3) some participants will also be assigned to identify social supporters to receive SMS reminders; and 4) other participants will not receive any SMS reminders.

If an individual agrees to participate, we will complete the screening process by driving the potential participant home to assess the adequacy of cellular phone reception. If adequate (i.e, three out of four bars or four out of five bars in at least one location), the research assistant will complete the consenting process by asking the participant to sign the consent form. A copy will be provided to the participant. Transport will be refunded in cases where the potential participants come purposely for the consenting process.

*Social supporters of the intervention study.* Study participants in the intervention arms (A and B) will be asked to identify potential social supporters at enrollment. We will then contact these individuals by cell phone to see if they are potentially interested in participating. If so, we will make arrangements for them coming to the study offices for screening and, if appropriate, consent. All activities will be conducted in a private space. Using the consent form and the language preferred by the patient, we will introduce the study and gauge the individual’s interest. Eligibility criteria will be reviewed per the social supporter screening CRF. Potential participants will be told the purpose of the study and the reasons they have been approached for participation. The study procedures will be explained, including the randomization process, as well as anticipated risks and benefits. If an individual agrees to participate, we will complete the consenting process by asking the participant to sign the consent form. A copy will be provided to the participant. Transport will be refunded in cases where the potential participants come purposely for the consenting process.

B.5.2 Outline who will deliver the above information and how?

The PI and the Research Assistant will deliver the above information. See section B.5.1 for how this will be done.

B.5.3 Please indicate how consent will be obtained, given local circumstances.

*Guidance notes:*

*In some societies, the concept of giving consent on an individual basis is unfamiliar. It may be necessary to obtain consent both at community and individual level.*

*Obtaining consent from minors requires both consent from the guardian and, where possible, the minor.*

See section B.5.1 above.

B.5.4 Are any inducements to be offered to either participants or the individuals who will be recruiting them? (e.g. improved patient care / cash) (please tick appropriate box)

**Yes** No

B.5.5 If yes, please give details:

Consistent with the local cultural expectations and previous studies, we will provide participants with a kilogram of sugar or some soap for each of the two interviews in this study. They will also be given 10,000 Ugandan Shillings (per trip; equivalent of ~$4) to cover transportation costs if they come to the research offices for an interview. Light refreshments may also be offered.

B.5.6 Outline any hidden constraints to consent.

*Guidance notes:*

*Examples where hidden constraints may be important include:*

- *situations where participants are employees of the investigator*
- *patients who may feel their care could be compromised if they do not consent to research initiated by their carers.*

No hidden constraints to consent are involved. Participants will be informed that their decision to be or not to be in the study will not in any way affect the health services they get from MRRH.

**SECTION C**

**RESPONSIBILITY**

C.1 Litigation:

In respect of any litigation which may result from this research

a) Who will provide compensation?

We do not anticipate any litigation.

(Please provide documentary evidence where appropriate.)

b) What insurance arrangements have been made by the applicant and his/her delegated assistants?

NA

(Please ensure that any professional indemnity insurance is logged with the Director's office)

C.2 **DECLARATION: TO BE SIGNED BY MAIN APPLICANT**

- I confirm that the details of this proposal are a true representation of the research to be undertaken.

- I will ensure that the research does not deviate from the protocol described.

- If significant protocol amendments are required as the research progresses, I will submit these to the Mbarara University Faculty Medicine Research Ethics Committee for approval.

- Where an appropriate mechanism exists, I undertake to seek additional local Ethical Approval in the country(ies) where the research is to be carried out.

I expect the project to commence on (Date): November 2016, and be completed by

(Date): 2021

________________________________________________________________________

Signed Date

**SECTION D**

**APPROVALS**

D.1 List research team and all collaborators.

(Please include all overseas collaborators and give their affiliations, qualifications and role in the study).

Dr. Conrad Muzoora, MD, MUST, (Project Mentor)

Dr. Jessica Haberer, MD, Harvard Medical School, (Project Mentor)

Dr. Luke Davis, MD, Yale School of Public Health (Project Mentor)

1. Musiimenta, A.Social & Institutional issues in the Adoption of School-based Technology-aided Sexual Health Education Program. *Online Journal of Public Health Informatics,* 2013, 5(2).
2. Musiimenta, A. A Controlled Before-After Evaluation a computer-based HIV/AIDS education on Students’ Sexual Behaviours, Knowledge and Attitudes. *Online Journal of Public Health Informatics*, 2012, 4(1).
3. Musiimenta, A. Information Technology-Mediated Issues in Sexual Health and HIV/AIDS Education. *Electronic Journal of Information Systems in Developing Countries*, 2012, 53(3).
4. Musiimenta, A. Contextual Mediators influencing the Effectiveness of Behavioural Change Interventions: A Case of HIV/AIDS Prevention Behaviours. *Online Journal of Public Health Informatics,* 2012, 4(2).
5. Campbell JI, Eyal N, Musiimenta A, Haberer JE. Ethical Questions in Electronic Adherence Monitoring. *JGIM*, *Journal of General Internal Medicine*; 2015.
6. McLeroy KR, Bibeau D, Steckler A, Glanz K. An ecological perspective on health promotion programs. Health Educ Q; 1888;15(4):351-77.
7. Venkatesh V, Morris MG, Davis GB, Davis FD (2003) User acceptance of information technology: Toward a unified view. MIS quarterly: 425-478.
8. Ministry of Health. The Uganda Tuberculosis Communication Strategy. 2010.
9. Wynne W, Richter S, Banura L, Kill W, Challenges in tuberculosis care in Western Uganda: Health care worker and patient perspectives. [International Journal of Africa Nursing Sciences](http://www.sciencedirect.com/science/journal/22141391). [Volume 1](http://www.sciencedirect.com/science/journal/22141391/1/supp/C), 2014, Pages 6–10
10. Nglazi MD, Bekker LG, Wood R, Hussey G, Wiysonge. Mobile phone text messaging for promoting adherence to anti-tuberculosis treatment:
    a systematic review. *BMC Infectious Diseases* 2013, 13:566
11. Liu. X, Lewis.J, and Zhang.H et al (2015). Effectiveness of Electronic Reminders to improve Medication Adherence in Tuberculosis Patients. A Cluster-Randomised Trial, *PLoS,* 12(9).
12. Iribarren S, Chirico C, Echevarrria M, Cardinali D: TextTB: a parallel design randomized control pilot study to evaluate acceptance and
    feasibility of a patient-driven mobile phone based intervention to support adherence to TB treatment. Journal of Mobile Technology in
    Medicine 2012, 1:23–24
13. Aday LA, Andersen RM. A framework for the study of access to medical care. *Health Serv Res* 1974;9(3):208-220.
14. *WHO, Global report for tuberculosis. 2014.*
15. Haynes RB1, McDonald H, Garg AX, Montague P et al.(2005) Interventions for helping patients to follow prescriptions for medications.
16. *Bassili, G. T. a. A., 2014. Global Tuberculosis Report, France: 2014*
17. Broomhead S, Mars M: Retrospective return on investment analysis of an electronic treatment adherence device piloted in the Northern Cape Province. Telemed J E Health 2012, 18:24–31
18. Bridges.org: Evaluation of the On Cue Compliance Service pilot: Testing the Use of SMS Reminders in the Treatment of Tuberculosis in Cape Town, South Africa. Cape Town. Cape Town: Bridges.org; 2005. <http://healthmarketinnovations.org/sites/> default/files/Evaluation%20of%20the%20On%20Cue%20Compliance%20Service%20Pilot.pdf
19. World Bank. (2009). Mobile phone subscriptions. "Mobile Marvels". Available at: h[ttp://www.economist.com/node/14483896](http://www.economist.com/node/14483896). Accessed: 29 Nov 2015.
20. Sabin LL BDM, Gill CJ, Zhong L, Vian T, Xie W, et al. Improving Adherence to Antiretroviral Therapy With Triggered Real-time Text Message Reminders: The China Adherence Through Technology Study. J Acquir Immune Defic Syndr 2015;69:551-9
21. Orrell C, Cohen K, Mauff K, Bangsberg DR, Maartens G, Wood R. A randomised controlled trial of real-time electronic adherence monitoring with text message dosing reminders in people starting first-line antiretroviral therapy. J Acquir Immune Defic Syndr. 2015.
22. Coker-Appiah DS, Akers AY, Banks B, Albritton T, Leniek K, Wynn M, Youmans SE, Parker D, Ellison A, Henderson S, Stith D, Council B, Oxendine-Pitt P, Corbie-Smith G. In their own voices: rural African American youth speak out about community-based HIV prevention interventions. *Prog Community Health Partners*. 2009 Winter;3(4):301-12
23. Sandelowski M. Sample size in qualitative research. *Res Nurse Health*. 1995 Apr;18(2):179-83.
24. Crabtree BF MW. *Doing Qualitative Research*. Thousand Oaks, CA: Sage Publications, Inc.; 1999
25. Glaser B Strauss A. *The Discovery of Grounded Theory: Strategies for Qualitative Research*. Chicago:
    Aldine; 1967.
26. Miles M HA. *Qualitative Data Analysis: An Expanded Sourcebook*. Thousand Oaks, CA: Sage
    Publications, Inc.; 1994
27. Cohen JA. A coefficient of agreement for nominal scales. *Educ Psychol Meas*. 1960;20:37-46
28. Landis JK Koch G. The measurement of observer agreement for categorical data. *Biometrics*. 1977
    Mar;33(1):159-74
29. Boyatzis R. *Transforming Qualitative Information: Thematic Analysis and Code Development*. Thousand Oaks, CA: Sage Publications, Inc.; 1998
30. Strauss A Corbin J. *Basics of qualitative research*. Thousand Oaks, CA: Sage Publications, Inc.; 1998.
31. Devers KJ. “How Will We Know ‘Good’ Qualitative Research When We See It? Beginning the Dialogue in Health Services Research.” Health Services Research. *Health Serv Res*. 1999 Dec;34(5 Pt 2):1153-88.
32. Bolton P, Wilk CM, Ndogoni L. Assessment of depression prevalence in rural Uganda using symptom and function criteria. Soc Psychiatry Psychiatr Epidemiol. 2004;39(6):442-7.
33. Tsai AC, Bangsberg DR, Frongillo EA, Hunt PW, Muzoora C, Martin JN, et al. Food insecurity, depression and the modifying role of social support among people living with HIV/AIDS in rural Uganda. Soc Sci Med. 2012;74(12):2012-9.
34. Bush K, Kivlahan DR, McDonell MB, Fihn SD, Bradley KA. The AUDIT alcohol consumption questions (AUDIT-C): an effective brief screening test for problem drinking. Ambulatory Care Quality Improvement Project (ACQUIP). Alcohol Use Disorders Identification Test. Arch Intern Med. 1998;158(16):1789-95.
35. Broadhead WE, Gehlbach SH, de Gruy FV, Kaplan BH. The Duke-UNC Functional Social Support Questionnaire. Measurement of social support in family medicine patients. Med Care. 1988;26(7):709-23.
36. Kalichman SC, Simbayi LC, Cloete A, Mthembu PP, Mkhonta RN, Ginindza T. Measuring AIDS stigmas in people living with HIV/AIDS: the Internalized AIDS-Related Stigma Scale. AIDS Care. 2009;21(1):87-93.
37. Onken LS, Blaine JD, Battjes R. *Behavioral therapy research: a conceptualization of a process*. In SW Henngler and R Amentos (Eds.), Innovative approaches from difficult to treat populations (pp. 477-485). Washington, DC: American Psychiatric Press.
38. Creswell JW. *Qualitative Inquiry and Research Design: Choosing Among Five Traditions*. Thousand Oaks, CA: Sage Publications, Inc.; 1999.
39. Crabtree BF MW. *Doing Qualitative Research*. Thousand Oaks, CA: Sage Publications, Inc.; 1999
40. Volmink, J, Garner, P. Directly observed therapy for treating tuberculosis. Cochrane Database Syst Rev 2007;4.
41. Ware NC, Idoko J, Kaaya S, et al. Explaining adherence success in sub-Saharan Africa: an ethnographic study. PLoS Med 2009;6:e11.
42. Lutge EE WC, Knight SE, Volmink J: . Material incentives and enablers in the management of tuberculosis. Cochrane Database Syst Rev 2012;1.
43. M'Imunya JM KT, Volmink J. Patient education and counselling for promoting adherence to treatment for tuberculosis. Cochrane Database Syst Rev 2012;5.
44. Squire, S. B., Obasi, A., & Nhlema-Simwaka, B. (2006). The global plan to stop TB: A unique opportunity to address poverty and the millennium development goals. Lancet, 367(9514), 955–957.
45. Haberer JE, Musiimenta A, Atukunda EC, Musinguzi N, Wyatt MA, Ware NC, Bangsberg DR. (2016). SMS reminders plus real-time adherence monitoring improve adherence to antiretroviral therapy in rural Uganda. *AIDS.*
46. Ware NC, Pisarski EE, Tam M, Wyatt MA, Atukunda EC, Musiimenta A, Bangsberg DR, Haberer JE. . (2016). The Meanings in the Messages: How SMS Reminders and Real-time Adherence Monitoring Promote Adherence to Antiretroviral Therapy in Rural Uganda. *AIDS*.
47. Esther C Atukunda, Angella Musiimenta, Nicholas Musinguzi, Monique A. Wyatt, Justus Ashaba, Norma C. Ware, Jessica E. Haberer. (2016). Understanding patterns of social support and their relationship to an ART adherence intervention among adults in rural southwestern Uganda. *AIDS and Behavior*
48. Michiel S, Atkins L, and West R. (2014). The Behavioral Change Wheel: A Guide to Designing Interventions. 1^st^ Ed. Great Britain, Silverback Publishing.
