# Supplementary material for: The feasibility, acceptability, and preliminary impact of real-time monitors and SMS on tuberculosis medication adherence in southwestern Uganda: Findings from a mixed methods pilot randomized controlled trial
Source: PLOS Glob Public Health. 2023 Dec 5;3(12):e0001813. doi: 10.1371/journal.pgph.0001813 (PMC10697590; doi:10.1371/journal.pgph.0001813)
Supplement: S1 Text — (DOC) [file pgph.0001813.s005.doc]

**Questionnaire packet—Study Title: Real time Tuberculosis medication Adherence intervention in Rural southwestern Uganda.**

Table Contents

[Demographics and health- Wisepill participant 2](#__RefHeading___Toc134809712)

[Cell phone use 3](#__RefHeading___Toc134809713)

[Socio-economic status 7](#__RefHeading___Toc134809714)

[Food security 8](#__RefHeading___Toc134809715)

[Depression 11](#__RefHeading___Toc134809716)

[Alcohol use 12](#__RefHeading___Toc134809717)

[Social support- Wisepill participant 13](#__RefHeading___Toc134809718)

[Social support: Social supporter 14](#__RefHeading___Toc134809719)

[Stigma- Wisepill participants 15](#__RefHeading___Toc134809720)

[Technology Adoption (Pre-Intervention) 16](#__RefHeading___Toc134809721)

[Technology Adoption (Post-Intervention) 19](#__RefHeading___Toc134809722)

[Exit interview- Wisepill participants (Arms A and B) 27](#__RefHeading___Toc134809723)

[Exit interview- Social supporters 31](#__RefHeading___Toc134809724)

# Demographics and health- Wisepill participant

Participant ID__ __ __ Date __ __/__ __/__ __ __ __

Staff initials __ __ __
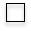
Enrollment
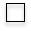
 Interim

Participant ID__ __ __ Date __ __/__ __/__ __ __ __

Staff initials __ __ __
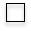
Enrollment
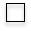
 Interim

Participant ID__ __ __ Date __ __/__ __/__ __ __ __

Staff initials __ __ __
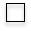
Enrollment
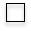
 Interim

*Instructions: To be completed at enrollment, referencing the participant’s medical chart in the TB Clinic as needed. This form may also be used to document changes during the study (complete with only new information).*

**Demographics**

1. Date of birth: __ __/__ __/__ __ __ __

*d d m m y y y y*

1. Gender: Male  Female

| 3. Primary residence | | | |
| --- | --- | --- | --- |
| County | Sub-County | Parish | Village/LC1 |
|  |  |  |  |
| 4. Would you call your residence a town or a rural area? | | | |
| Town | Rural |  | |

1. Marital Status

Married Single  Others Please specify

1. Highest level of education achieved:

None P1-P7 >P7

1. Literacy

|  | English | Runyankole |
| --- | --- | --- |
| Able to speak | Yes No | Yes No |
| Able to read (*refer to language card*) | Yes No | Yes No |

# Cell phone use

*Instructions: To be completed at enrollment and any time this information changes (complete with only new information).*

| 1. Preferred cell phone number | ___ ___ ___ ___ ___ ___ ___ ___ ___ ___ |
| --- | --- |
| 2. Alternate cell phone numbers  (check here if none  ) | ___ ___ ___ ___ ___ ___ ___ ___ ___ ___  ___ ___ ___ ___ ___ ___ ___ ___ ___ ___ |

1. Who else uses your telephone?

None Spouse  Other family member  Friend  Neighbor

Other (please specify)-----------------------------------------*Instructions:*

***Complete the remainder of this form for participants in the intervention arms only.***

1. How often do you check your phone for SMS messages in a typical week?

None  1 - 3 times  4 -7 times  > 7

1. Have you ever been delayed from your usual schedule of checking your SMS messages because of any of the following in the last week:

Your phone wasn’t charged?

Your SIM card wasn’t in your phone?

You did not have adequate cell signal?

Your phone was not functioning?

Someone else (i.e. family member who you share the phone with) had your phone at the time?

Any other reason why you were unable to check your phone for SMS messages?

1. Do you ever use your phone for anything related to your health?

Yes  No

<If yes>

1. What mode was the conversation? (tick all that applies

SMS texts  Voice calls

- 1. What did you use your phone for? (tick all that applies)

Arranging transportation/visit to the clinic

Helping take medication including inquiring about medication symptoms

Other, please specify………………………………………...................

1. What content of SMS messages would you like?

Would you prefer a direct SMS reminder (e.g. ‘X, please remember your TB medication’) or

Would you prefer an indirect one (e.g. ‘this is your reminder’)?

Please give reasons for your preference.………………………………………………

………………………………………………………………………………………….

………………………………………………………………………………………….

………………………………………………………………………………………….

………………………………………………………………………………………….

1. Language preference for SMS: Runyankole English

***Instructions: For participants in Intervention Arm A only, ask questions 13 and 14. Otherwise, skip to question 15.***Preferred time for SMS: __ __:__ __ (use 24 hour clock)

1. Preferred day for weekly SMS:

Sunday Monday Tuesday Wednesday Thursday

Friday Saturday

Preferred content for the SMS (max 140 characters).*Wisepill participants not wishing to personalize content will receive the message, “This is your reminder”. Add additional pages as needed.*---------------------------------------------------------------------------------------------------------------

Which barriers do you anticipate to experience in receiving SMS reminders for TB medication?

Charging the phone

Message being seen by people you are not comfortable with

Other (Specify)

**Health (check the screening form for 15 and 16)**

1. **Tuberculosis**
2. TB medication regimen (from clinical records)

isoniazid  rifampicin  pyrazinamide  ethambutol  Other

1. Type of TB infection

pulmonary,  other

1. Date of initiating TB treating_____________________
2. Duration of TB treatment planned______________________________
3. Ever disclosed you TB Status to anyone other than healthcare provider?

Yes No

1. **HIV/AIDS** (Check on the medical chart)
2. HIV Status Negative Positive
3. <if positive> Date of initiating treatment____________
4. <*for a patient*> I find it easier to take TB drugs than ARTs

Strongly agree  Agree  Disagree  Strongly disagree

**Social Support**

1. Your social supporter’s preferred telephone number:-------------------------------
2. Your social supporter’s alternate telephone number:--------------------------------
3. Relationship with your social support: (e.g. spouse)-------------------------------------
4. Why have you chosen that social supporter? ------------------------------------------------------------------------------------------------------------------------------------------------------------------------------------------------------------------------------------
5. How many times in the past week has your social supporter provided you with support?

None  1 - 3 times  4 -7 times  > 7

1. If you received support in the past week, what kind of support was it? (tick all that applies)

Transport to clinic  helping with chores  helping with childcare  reminders/encouragement to take medication  Other (please specify) ……………………………….

1. Do you feel that the support s/he provided was enough for you (up to your expectations)?

Yes  No

If no, why?.........................................................................

**TB Medication Taking Behavior**

1. Which of the following is true about your TB medication taking behavior?

Often take pills on the exact time prescribed by the Doctor?

Sometimes delays taking medication

Sometimes misses taking pills

Pease give reasons for your answer……………………………………………

1. Which of the following explains why you sometimes miss or delay taking your medication? Tick all that apply.

Forgets that it is time to take medication

Lacks transport to pick medication from the clinic

I don’t think taking medication on time is important

others (please specify)

1. Do you think that being in a study that sends you SMS reminders to take your TB medication would help you take your medication in time?

Yes  No

If yes, why…………………………………………..

1. Do you think that giving you a device that monitors and reports to us how you take your medication would help you take your medication in time?

Yes  No

If yes, why…………………………………………..

1. In case you miss your dose, do you think that notifying someone who provides health-related support to you (e.g. someone who provides transport to clinic, takes care of chores when you go to clinic) would help you take your medication in time?

Yes  No

If yes, why……………………………………

# Socio-economic status

**Read:** Now I will ask you some questions about your household. For this study, consider your household to include people with whom you usually live and share meals. Please include children who are in boarding school or who are sent to live with relatives elsewhere. If you live in your employer's household, refer to you own family's household. Some questions are meant for people living in rural areas, while others are meant for people living in urban areas. If a question does not apply to you, just let me know.

*RA instructions: If a participant resides in multiple households, prompt for the primary or main household she/he lives in, defined by where she/he spends the majority of time. If no primary household can be identified, choose where she/he most recently resided.*

1. In your household:

1. Is there running water?: Y  N
2. Is there a non-dirt floor?: Y  N
3. Is there electricity?: Y  N
4. Is the roof made of tile, or metal such as iron, tin, or zinc?: Y  N
5. How many rooms (i.e., living, dining, bedroom, bathroom, kitchen) are in the house?:|_____|
6. How many people live in the house?:|_____|

2. How did you come to live in your current home?

Rent  Bought (self or spouse)  Inherited

Moved in with family/friends Other (*specify* ________________)

**Read:** Next, I would like to ask you about economic and social activities. We are interested in learning about different kinds of activities, including not only regular work but also petty jobs and occasional work such as selling firewood or household items.

3. Do you yourself have a regular income? By this I mean fixed wages or a fixed salary that you receive on a regular basis, for example every two weeks or every month, and is not dependent on the seasons or on how much you work.

Yes  No -> #4

***If YES:***

3a. Approximately how much money did you earn last month as regular income?

__,__ __ __,__ __ __ USh

4. Do you have any income from other sources (e.g., casual labor, handouts from a friend or relative)?

***If YES:***

4a. Approximately how much money did you get last month from other incomesources?

__,__ __ __,__ __ __ USh

**Read:** We would like to know more about your household spending, so now I will ask you questions about things like food, livestock, health care, and transport. We will not be providing any of the items that will be mentioned, so you may answer freely. I will ask you how much money your household spend in these categories in the past month. "Household" means all of the people who stay in the same house and eat meals together.

| SPENDING TYPE | In the past 1 month, how much money did your household spend on purchasing ***[TYPE]***? |
| --- | --- |
| -Food or water to eat or drink | __,__ __ __,__ __ __ USh |
| -Livestock, including cows, pigs, goats, sheep, chickens, or any other kind of animal | __,__ __ __,__ __ __ USh |
| -Supplies for your garden, including seedlings, manure, or tools (but not including the land) | __,__ __ __,__ __ __ USh |
| -Rent and utilities | __,__ __ __,__ __ __ USh |
| -Health care, including modern medicines, fees for doctor or hospital visits, fees for the traditional healer, or transport to the hospital or clinic | __,__ __ __,__ __ __ USh |
| -Transport (not including money spent on transport to the hospital or clinic) | __,__ __ __,__ __ __ USh |
| -Expenses for public gatherings, including weddings, religious festivals, parties, or burials | __,__ __ __,__ __ __ USh |
| -School fees | __,__ __ __,__ __ __ USh |
| -Other household matters, like clothes or improvements to the structure of your home | __,__ __ __,__ __ __ USh |

# Food security

*Instructions: To be completed by a study RA at enrollment.*

The following questions ask about what you typically eat. Please note that while this research will be used to guide future programs and policies, we will NOT be distributing food items during or after this research project. Please answer these questions as truthfully as possible so that we can accurately assess food availability in this area. Whenever we say "lack of resources", we mean not having money, land, or manpower. "Household" means all of the people who stay in the same house and eat meals together.

*For each of the following questions, consider what has happened in the past 30 days. Please answer whether this happened never; rarely, meaning once or twice; sometimes, meaning 3-10 times; or often, meaning more than 10 times in the past 30 days.*

|  | Never | Rarely | Sometimes | Often |
| --- | --- | --- | --- | --- |
| 1. Did you worry that your household would not have enough food? |  |  |  |  |
| 2. Were you or any household member not able to eat the kinds of foods you preferred because of a lack of resources? Foods you preferred might include meat, poultry, eggs, fish, milk, matooke, spaghetti and karo. |  |  |  |  |
| 3. Did you or any household member eat just a few kinds of food, such as only matooke or posho with either beans, groundnuts or dodo, day after day due to a lack of resources? |  |  |  |  |
| 4. Did you or any household member eat food that you preferred not to eat because of a lack of resources to obtain other types of food? A food you preferred not to eat might include cassava, matooke flour, posho, embile, dodo, katunkuma, and entura. |  |  |  |  |
| 5. Did you or any household member have to eat less at any meal than you felt you needed to eat because there was not enough food? |  |  |  |  |
| 6. Did you or any household member eat fewer meals in a day because there was not enough food? |  |  |  |  |
| 7. Was there ever no food at all in your household granaries or gardens because there were not resources to get more? |  |  |  |  |
| 8. Did you or any household member go to sleep at night hungry because there was not enough food? |  |  |  |  |
| 9. Did you or any household member go a whole day without eating anything because there was not enough food? |  |  |  |  |

Participant ID__ __ __ Date __ __/__ __/__ __ __ __

Staff initials __ __ __

**Food Security**

10. At any time during the past year did you or your family receive food aid? By food aid I mean free food that you got from church, clinic, non-governmental organizations, or governmental organizations? I don't mean food that you got from family or friends.

Yes No ---> form is complete

11. Where did you get food aid from? Mark all that apply:

Church Non-governmental organization

Clinic Governmental organization

Other (specify): ___________________________________________________

12. Did you or your family receive food aid over the past 30 days?

Yes No ---> form is complete

13. Over the past 30 days, how many days did you eat free food that you got from church, clinic, non-governmental organizations, or governmental organizations? I don't mean food that you got from family or friends.

__ __ # of days

Participant ID__ __ __ Date __ __/__ __/__ __ __ __

Staff initials __ __ __

# Depression

*Instructions:* The next set of questions is a list of problems that people can get. You can take this part yourself or I can read it to you, whatever you are most comfortable with. These questions are about how you have been feeling during the past week.

*If the participant chooses to self-administer*: Fill in the circle that BEST describes the way you've been feeling during the past week. Please feel free to ask any questions and take as much time as you need.

*If the participant chooses to have questions read to him/her*: For each item I will read to you, please let me know if you have felt this or experienced this not at all; a little; quite a bit; or extremely.

|  | Not at all | A little | Quite a bit | Extremely |
| --- | --- | --- | --- | --- |
| 1. Feeling low in energy, slowed down |  |  |  |  |
| 2. Blaming yourself for things |  |  |  |  |
| 3. Crying easily |  |  |  |  |
| 4. Feeling fidgety |  |  |  |  |
| 5. Poor appetite |  |  |  |  |
| 6. Difficulty falling asleep or staying asleep |  |  |  |  |
| 7. Feeling hopeless about the future |  |  |  |  |
| 8. Feeling sad |  |  |  |  |
| 9. Feeling lonely |  |  |  |  |
| 10. Thoughts of ending your life |  |  |  |  |
| 11. Worrying too much about things |  |  |  |  |
| 12. Feeling no interest in things |  |  |  |  |
| 13. Feeling everything is an effort |  |  |  |  |
| 14. Feeling of worthlessness |  |  |  |  |
| 15. Loss of sexual interest or sexual pleasure |  |  |  |  |
| 16. Feeling like I don't care what happens to my health |  |  |  |  |

Participant ID__ __ __ Date __ __/__ __/__ __ __ __

Staff initials __ __ __

# Alcohol use

*Instructions: Please read the following. For each question, read the potential answer choices.*

Now I have some questions about alcohol. Because alcohol use can affect many areas of health, it is important for us to know how much you drink. Please try to be as honest and accurate as possible. It is important in assessing your health for us to know what you actually do.

Alcohol includes wine, beer, or hard liquor, [insert other local terms for alcoholic beverage], or any beverage that contains alcohol. Please do not include communion wine or wine that you received at church or a religious ceremony when answering these questions.

1. How often do you have a drink containing alcohol?

Never

Monthly or less

2-4 times a month

2-3 times a week

4 or more times a week

2. How many standard drinks containing alcohol do you have on a typical day

1 or 2

3 or 4

5 or 6

7 or 9

10 or more

3. How often do you have six or more drinks on one accession?

Never

Less than a month

Monthly

Weekly

Daily or almost daily

Participant ID__ __ __ Date __ __/__ __/__ __ __ __

Staff initials __ __ __
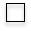
Baseline
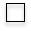
Follow-up

# Social support- Wisepill participant

*Instructions: To be completed at enrollment and at the second interview.*

I am going to ask you some questions about your social support. Thinking about your current situation, for each question you may answer, 'as much as I would like', 'less than I would like', 'much less than I would like', or 'never'. I will then ask you for the number of people who provide you with each type of support.

|  | As much as I would like | Less than I would like | Much less than I would like | Never |
| --- | --- | --- | --- | --- |
| 1. I get visits from friends and relatives |  |  |  |  |
| 2. I get useful advice about important things in my life |  |  |  |  |
| 3. I get chances to talk to someone about problems at work or with my housework |  |  |  |  |
| 4. I get chances to talk to someone I trust about my personal and family problems |  |  |  |  |
| 5. I have people who care what happens to me |  |  |  |  |
| 6. I get love and affection |  |  |  |  |
| 7. I get help with household-related work |  |  |  |  |
| 8. I get help with money in an emergency |  |  |  |  |
| 9. I get help when I need transportation |  |  |  |  |
| 10. I get help when I am sick |  |  |  |  |

11. In total, how many different people provide you with any of the above social support? __ __

12. Are you a member of a TB support group or post-test club?

Yes No

Participant ID__ __ __ Date __ __/__ __/__ __ __ __

Staff initials __ __ __

# Social support: Social supporter

|  | As much as I would like | Less than I would like | Much less than I would like | Never |
| --- | --- | --- | --- | --- |
| 13. I visit friends and relatives |  |  |  |  |
| 14. I give useful advice about important things in others' lives |  |  |  |  |
| 15. I talk to others about problems at their work or with their housework |  |  |  |  |
| 16. I talk to others about their personal and family problems |  |  |  |  |
| 17. I care what happens to people in my life |  |  |  |  |
| 18. I give love and affection |  |  |  |  |
| 19. I give help with household-related work |  |  |  |  |
| 20. I give help with money in an emergency |  |  |  |  |
| 21. I give help when others need transportation |  |  |  |  |
| 22. I give help when others are sick |  |  |  |  |

Now I am going to ask you the same questions about the social support you provide for others. Thinking about your current situation, for each question you may answer, 'as much as I would like', 'less than I would like', 'much less than I would like', or 'never'. I will then ask you for the number of people to whom you provide each type of support.

|  | All of the time | A lot of the time | Some of the time | Not at all |
| --- | --- | --- | --- | --- |
| 23. My family, friends, and/or community rely on me financially |  |  |  |  |
| | 24. My family, friends, and/or community rely on  me emotionally |  |  |  |  | | --- | --- | --- | --- | --- | |  |  |  |  |

The next set of questions also asks about the social support you provide for others. Thinking about your current situation, for each question you may answer, 'all of the time', 'a lot of the time’, ‘some of the time’, or ‘not at all’.

Participant ID__ __ __ Date __ __/__ __/__ __ __ __

Staff initials __ __ __ Baseline Follow-up

# Stigma- Wisepill participants

*Instructions: To be completed by at enrollment and at the second interview.*

Please tell me if you either agree or disagree with each of these statements:

|  | Agree | Disagree |
| --- | --- | --- |
| 1. It is difficult to tell other people about my TB infection. |  |  |
| 2. Having TB makes me feel immoral. |  |  |
| 3. I feel guilty that I am I have TB. |  |  |
| 4. I am ashamed that I have TB. |  |  |
| 5. I sometimes feel worthless because I have TB. |  |  |
| 6. It is my own fault that I have TB. |  |  |
| 7. I hide my TB status from others. |  |  |
| 8. I feel certain that I can tell my sex partner that I have TB |  |  |

9. Have you told anyone that you have TB, not including your health provider?

Yes No 
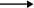
 Form is complete

Who have you told that you that you have TB?

|  | Yes | No | N/A |
| --- | --- | --- | --- |
| Spouse? |  |  |  |
| Sexual partner? |  |  |  |
| Family member? |  |  |  |
| Friend? |  |  |  |
| Neighbor? |  |  |  |
| Workplace colleague? |  |  |  |
| Religious leader? |  |  |  |
| Anyone else? |  |  |  |
| If yes, specify ______________________________________________ | | | |

Participant ID__ __ __ Date __ __/__ __/__ __ __ __

Staff initials __ __ __

10. How many people in the home you usually live in know that you have TB? Would you say that it is no one, a few of the people, half of the people, most of the people, or everyone?

No one A few of the people Half of the people

Most of the people Everyone

# Technology Adoption (Pre-Intervention)

**Study Title: Real time Tuberculosis medication Adherence intervention in Rural southwestern Uganda.**

Hope you are fine. We are conducting a study whose purpose is to learn how to support people like you who are taking TB medications using SMS (text messages) and a special pill container called Wisepill. This pill container sends us an electronic signal over cellular phone networks to let us know someone opened it to take his/her medication. Here is a Wisepill device that we will give to Wisepill participants <*practically show how the wisepill works including how to open, close, charge it, and how it sends signals*. We will send you SMS reminders take their medications. We will also plan send SMS notifications to your Social supporters who have provided you with help in the past (for instance, to get to clinic to see the doctor) and know they have TB. We hope to learn if either the SMS reminders directly to you or the SMS notifications to your Social supporters will help you take your medications. The following questions will help us understand opinions about using the Wisepill container, SMS reminders for the Wisepill participants, and SMS notifications to Social supporters to encourage people take TB medications.

**Perceived Usefulness of the wisepill device**

1. Using the wisepill device will be useful in my TB medication adherence.

❑Strongly Agree ❑Agree ❑Disagree ❑Strongly Disagree

1. Using the wisepill device will positively affect the way I feel about taking my TB medicine. ❑Strongly Agree ❑Agree ❑Disagree ❑Strongly Disagree.
2. Using the wisepill device will help me take my TB medication in time/as prescribed. ❑Strongly Agree ❑Agree ❑Disagree ❑Strongly Disagree.
3. Using the wisepill device will make it easier to take my TB medication.

❑Strongly Agree ❑Agree ❑Disagree ❑Strongly Disagree.

**Perceived Usefulness of using the SMS reminders**

1. Using SMS reminders will be useful in my TB medication adherence.

❑Strongly Agree ❑Agree ❑Disagree ❑Strongly Disagree

1. Using SMS reminders will positively affect the way I feel about taking my TB medicine. ❑Strongly Agree ❑Agree ❑Disagree ❑Strongly Disagree.
2. Using SMS reminders will help me take my TB medication in time/as prescribed. ❑Strongly Agree ❑Agree ❑Disagree ❑Strongly Disagree.
3. Using the SMS reminders will make it easier to take my TB medication.

❑Strongly Agree ❑Agree ❑Disagree ❑Strongly Disagree.

**Perceived Usefulness of SMS notifications for social supporters (for participants in Arms A and B only)**

1. Sending SMS notifications to my social supporter would be useful in my TB medication adherence. ❑Strongly Agree ❑Agree ❑Disagree ❑Strongly Disagree.
2. Sending SMS notifications to my social supporter would help me take my TB medication in time/as prescribed. ❑Strongly Agree ❑Agree ❑Disagree ❑Strongly Disagree.
3. Sending SMS notifications to my social supporter would positively affect the way I feel about taking my TB medicine. ❑Strongly Agree ❑Agree ❑Disagree ❑Strongly Disagree.
4. Sending SMS notifications to my social supporter would make it easier to take my TB medication. ❑Strongly Agree ❑Agree ❑Disagree ❑Strongly Disagree.

# Technology Adoption (Post-Intervention)

**(Arm A and B)**

**Study Title: Real time Tuberculosis medication Adherence intervention in Rural southwestern Uganda.**

Hope you are fine. You have been participating in a study that utilized the wisepill container, SMS reminders, and SMS notifications to social supporters. The following questions are meant to help us understand your experience of using the intervention.

**Perceived Usefulness of the wisepill device**

1. Using the wisepill device was useful in my TB medication adherence.

❑Strongly Agree ❑Agree ❑Disagree ❑Strongly Disagree

1. Using the wisepill device was more useful than the current pill containers used for TB medicines. ❑Strongly Agree ❑Agree ❑Disagree ❑Strongly Disagree.
2. Using the wisepill device positively affected the way I feel about taking my TB medicine. ❑Strongly Agree ❑Agree ❑Disagree ❑Strongly Disagree.
3. Using the wisepill device helped me take my TB medication in time/as prescribed. ❑Strongly Agree ❑Agree ❑Disagree ❑Strongly Disagree.
4. Using the wisepill device made it easier to take my TB medication.

❑Strongly Agree ❑Agree ❑Disagree ❑Strongly Disagree.

**Perceived Usefulness of using the SMS reminders**

1. Using SMS reminders was useful in my TB medication adherence.

❑Strongly Agree ❑Agree ❑Disagree ❑Strongly Disagree

1. Using SMS reminders positively affected the way I feel about taking my TB medicine. ❑Strongly Agree ❑Agree ❑Disagree ❑Strongly Disagree.
2. Using SMS reminders helped me take my TB medication in time/as prescribed. ❑Strongly Agree ❑Agree ❑Disagree ❑Strongly Disagree.
3. Using the SMS reminders made it easier to take my TB medication.

❑Strongly Agree ❑Agree ❑Disagree ❑Strongly Disagree.

**Perceived Usefulness of SMS notifications for social supporters**

1. Sending SMS notifications to my social supporter was useful in my TB medication adherence. ❑Strongly Agree ❑Agree ❑Disagree ❑Strongly Disagree.
2. Sending SMS notifications to my social supporter helped me take my TB medication in time/as prescribed. ❑Strongly Agree ❑Agree ❑Disagree ❑Strongly Disagree.
3. Sending SMS notifications to my social supporter positively affected the way I feel about taking my TB medicine. ❑Strongly Agree ❑Agree ❑Disagree

❑Strongly Disagree.

1. Sending SMS notifications to my social supporter made it easier to take my TB medication. ❑Strongly Agree ❑Agree ❑Disagree ❑Strongly Disagree.

**Perceived Ease of Use of the Wisepill Device**

1. It was easy for me to open the wisepill device to take my TB medication.

❑Strongly Agree ❑Agree ❑Disagree ❑Strongly Disagree.

1. It was easy for me to remember how to get TB pills from the wisepill device.

❑Strongly Agree ❑Agree ❑Disagree ❑Strongly Disagree.

1. It was easy for me to charge the wisepill device. ❑Strongly Agree ❑Agree ❑Disagree ❑Strongly Disagree.
2. It was easy for me to travel with the wisepill device. ❑Strongly Agree ❑Agree ❑Disagree ❑Strongly Disagree.

**Perceived Ease of Use of SMS Reminders for taking TB Medication**

1. It was easy for me to learn how to access the SMS reminders for taking my TB medication. ❑Strongly Agree ❑Agree ❑Disagree ❑Strongly Disagree.
2. It was easy for me to read the SMS reminders for taking my TB medication.
3. It was easy for me to understand/remember the SMS reminders for taking my TB medication. ❑Strongly Agree ❑Agree ❑Disagree ❑Strongly Disagree.
4. It was easy for me to remember to take my TB medication after receiving the SMS reminders. ❑Strongly Agree ❑Agree ❑Disagree ❑Strongly Disagree.

**Social Norms about using the Wisepill Device for my TB Medication**

1. People who take care of my health thought I should use the wisepill device for my TB medication. ❑Strongly Agree ❑Agree ❑Disagree ❑Strongly Disagree.
2. People who influence my behaviour thought I should use the wisepill device for my TB medication. ❑Strongly Agree ❑Agree ❑Disagree ❑Strongly Disagree.
3. People who are important to me thought I should use the wisepill device for my TB medication. ❑Strongly Agree ❑Agree ❑Disagree ❑Strongly Disagree.
4. People who use the wisepill device have high profile than those who do not use the device for my TB medication. ❑Strongly Agree ❑Agree ❑Disagree ❑Strongly Disagree.

**Social Norms about using SMS reminders for TB medication**

1. People who take care of my health thought I should use the SMS reminders for my TB medication. ❑Strongly Agree ❑Agree ❑Disagree ❑Strongly Disagree.
2. People who influence my behaviour thought I should use the SMS reminders for my TB medication. ❑Strongly Agree ❑Agree ❑Disagree ❑Strongly Disagree.
3. People who are important to me thought I should use the SMS reminders for my TB medication. ❑Strongly Agree ❑Agree ❑Disagree ❑Strongly Disagree.

**Facilitating Conditions**:

1. I posse a personal mobile telephone needed to received SMS reminders. ❑Strongly Agree ❑Agree ❑Disagree ❑Strongly Disagree.
2. My personal mobile phone successfully receives SMS reminders.❑Strongly Agree ❑Agree ❑Disagree ❑Strongly Disagree.
3. I have electricity/solar for charging my mobile phone. ❑Strongly Agree ❑Agree ❑Disagree ❑Strongly Disagree.
4. I have reliable mobile telephone network at my home that could enable me receive SMS reminders. ❑Strongly Agree ❑Agree ❑Disagree ❑Strongly Disagree.

**Using the wisepill device**

I used the wisepill device consistently. ❑Strongly Agree ❑Agree ❑Disagree

❑Strongly Disagree.

**Using the SMS reminders**

I used the SMS reminders consistently.

❑Strongly Agree ❑Agree ❑Disagree ❑Strongly Disagree.

**Acceptance of social support intervention**

It was good to have SMS notifications sent to my predefined social supporter when I missed taking my TB medication for 48 hours?

❑Strongly Agree ❑Agree ❑Disagree ❑Strongly Disagree.

**Ethical issues**

❑ Researchers decided to use the Wisepill device to monitor how I am taking my TB since they cannot trust me if I tell them that I am taking my medication on time.

❑I have some worries that researchers may not keep my status and TB adherence information to be generated by the device totally private.

❑Even when I was in public, I felt comfortable to read the SMS reminders, or open the device to take my TB medication.

❑ Using the device/SMS reminders exposed my TB status to people I am not comfortable knowing my status.

❑ After using the device/SMS reminders to support my TB medication taking for some time, I can still take my TB medication on time even if the device/SMS reminders are taken away.

**Perceived Ease of Use of the Wisepill Device**

1. It would be easy for me to open the wisepill device to take my TB medication.

❑Strongly Agree ❑Agree ❑Disagree ❑Strongly Disagree.

1. It would be easy for me to remember how to get TB pills from the wisepill device.

❑Strongly Agree ❑Agree ❑Disagree ❑Strongly Disagree.

1. It would be easy for me to charge the wisepill device. ❑Strongly Agree ❑Agree ❑Disagree ❑Strongly Disagree.
2. It will be easy for me to travel with the wisepill device. ❑Strongly Agree ❑Agree ❑Disagree ❑Strongly Disagree.

**Perceived Ease of Use of SMS Reminders for taking TB Medication**

1. It would be easy for me to learn how to access the SMS reminders for taking my TB medication. ❑Strongly Agree ❑Agree ❑Disagree ❑Strongly Disagree.
2. It would be easy for me to read the SMS reminders for taking my TB medication.

❑Strongly Agree ❑Agree ❑Disagree ❑Strongly Disagree.

1. It would be easy for me to understand/remember the SMS reminders for taking my TB medication. ❑Strongly Agree ❑Agree ❑Disagree ❑Strongly Disagree.
2. It will be easy for me to remember to take my TB medication after receiving the SMS reminders. ❑Strongly Agree ❑Agree ❑Disagree ❑Strongly Disagree.

**Social Norms about using the Wisepill Device for my TB Medication**

1. People who take care of my health will think I should use the wisepill device for my TB medication. ❑Strongly Agree ❑Agree ❑Disagree ❑Strongly Disagree.
2. People who influence my behaviour will think I should use the wisepill device for my TB medication. ❑Strongly Agree ❑Agree ❑Disagree ❑Strongly Disagree.
3. People who are important to me think will think I should use the wisepill device for my TB medication. ❑Strongly Agree ❑Agree ❑Disagree ❑Strongly Disagree.
4. People who use the wisepill device have high profile than those who do not use the device for my TB medication. ❑Strongly Agree ❑Agree ❑Disagree ❑Strongly Disagree.

**Social Norms about using SMS reminders for TB medication**

1. People who take care of my health think I should use the SMS reminders for my TB medication. ❑Strongly Agree ❑Agree ❑Disagree ❑Strongly Disagree.
2. People who influence my behaviour think I should use the SMS reminders for my TB medication. ❑Strongly Agree ❑Agree ❑Disagree ❑Strongly Disagree.
3. People who are important to me think I should use the SMS reminders for my TB medication. ❑Strongly Agree ❑Agree ❑Disagree ❑Strongly Disagree.

**Facilitating Conditions**:

1. I possess a personal mobile telephone needed to received SMS reminders. ❑Strongly Agree ❑Agree ❑Disagree ❑Strongly Disagree.
2. My personal mobile phone successfully receives SMS reminders.❑Strongly Agree ❑Agree ❑Disagree ❑Strongly Disagree.
3. I have electricity/solar for charging my mobile phone. ❑Strongly Agree ❑Agree ❑Disagree ❑Strongly Disagree.
4. I have reliable mobile telephone network at my home that could enable me receive SMS reminders. ❑Strongly Agree ❑Agree ❑Disagree ❑Strongly Disagree.

**Intentions to use**

- - - 1. Given an opportunity, I could use the Wisepill device. ❑Strongly Agree ❑Agree ❑Disagree ❑Strongly Disagree.
      2. Given an opportunity, I could use the SMS reminders.

❑Strongly Agree ❑Agree ❑Disagree ❑Strongly Disagree.

- - - 1. Given an opportunity, I intend to accept that SMS notifications be sent to my predefined social supporter if I miss taking my TB medication for 48 hours?

❑Strongly Agree ❑Agree ❑Disagree ❑Strongly Disagree.

**Ethical issues**

1. Which of the following is true about you (tick all that applies)

Researchers decide to use the Wisepill device to monitor how I am taking my TB since they cannot trust me if I tell them that I am taking my medication on time.

❑Strongly Agree ❑Agree ❑Disagree ❑Strongly Disagree.

I have some worries that researchers may not keep my status and TB adherence information to be generated by the device totally private.

❑Strongly Agree ❑Agree ❑Disagree ❑Strongly Disagree.

Even when I am in public, I can feel comfortable to read the SMS reminders, or open the device and take my TB medication.

❑Strongly Agree ❑Agree ❑Disagree ❑Strongly Disagree.

Using the device/SMS reminders can expose my TB status to people I am not comfortable knowing my status.

❑Strongly Agree ❑Agree ❑Disagree ❑Strongly Disagree.

After using the device/SMS reminders to support my TB medication taking for some time, I can still take my TB medication on time even if the device/SMS reminders are taken away.

❑Strongly Agree ❑Agree ❑Disagree ❑Strongly Disagree.

Participant ID__ __ __ Date __ __/__ __/__ __ __ __

Staff initials __ __ __

# Exit interview- Wisepill participants (Arms A and B)

*Instructions: Complete this form at disenrollment from the study.*

Was at least one SMS notification sent to the participant’s social supporter(s)?

Yes No --> skip to question 1d

Did your social supporter(s) contact you?

Yes No Don’t remember

Did your social supporter(s) provide you with help?

No (why?)_____

Yes (what kind of help?)

- Money
- Ride to clinic__
- Encouragement to take medication
- Counselling
- Other (specify)___________

<*If yes help was received>*

1b. How did you feel about receiving help from your social supporter in this way?

Very much disliked Disliked Liked Very much liked

1c. How interested would you be in continuing to receive support in this way?

Not interested at all Somewhat interested Very interested

1d*. If no or the participant was not contacted after all SMS notifications, ask:*

Why do you think you were not contacted after the SMS notification(s)? (*tick all that apply*)

My phone may have been turned off

My phone may not have had power

I switched to a new phone number

The network may not have been available

My social supporter(s) may have been too busy

My social supporter(s) may not have wanted to contact me

I have no idea

Other (specify) _______________________________________________

______________________________________________________________

2. Are you aware of any times when you did not open your Wisepill pill container for more than 48 hours and your social supporter did not contact you?

Yes No --> skip to question 3

2a.*If yes,* please explain the circumstances.__________________________________

_____________________________________________________________________

3. Did you receive any SMS reminders for not opening your Wisepill pill container within two hours of the time you said you were planning to take your medication?

Yes No --> skip to the end

3a. How did you feel about receiving the SMS reminders?

Very much disliked Disliked Liked Very much liked

Why?__________________________________________________________

3b. How interested would you be in continuing to receive SMS reminders?

Not interested at all Somewhat interested Very interested

Why?__________________________________________________________

3b. Which of the following describes you (tick as many as applicable)?

*For those who received daily SMS*

I preferred daily SMS reminders to SMS reminders that were coming after I have missed my medication

I preferred SMS reminders that were coming after I have missed my medication than daily SMS reminders.

Though I received daily SMS reminders, I would have preferred to receive weekly reminders

Please give reasons for your answer………………………………………………………….

*For those who received weekly SMS*

I preferred SMS reminders that were coming after I have missed my medication than weekly SMS reminders.

I preferred weekly SMS reminders to SMS reminders that were coming after I have missed my medication

Though I received weekly SMS reminders, I would have preferred to receive daily reminders

Please give reasons for your answer………………………………………………………….

3c.- Which of the following describes the type of SMS reminder you preferred to receive

The default SMS text (i.e. “T*his is your reminder”*)

Personalized SMS (i.e. reminders formulated by you e.g. “take care”)

4. How is your relationship with your social supporter now compared to how it was when you started the study?

*greatly improved* *slightly improved* *No change* *Slightly declined*

*Greatly declined*

Give reasons to support your answer____________________________________________________

5. Do you think the presence of support/or lack of support from your social supporter has been helpful and encouraging in any way? *Yes* *No*

Please explain_________________________________________________________________________________

(interviewer may probe for a detailed response).

6. What do you think motivated your social supporter to help or not to help you? _____________________________________________________________________________________________________

7. Do you sometimes worry that may social supporter may not keep the information about your TB status private? *Strongly* disagree *Disagree* *Agree* *Strongly agree*

8. How often do you communicate, visit or discuss with social supporter?

*At least daily* *Several times a week* *Once a week* *Once in 2 weeks* *Once a month* *Over a month* *Never*

9. Given another opportunity to participate in a related study, would you choose the same social supporter? Why or why not

10. Is there anything else you would like to tell me about the study?

Thank you. You have now completed your participation in this study.

Participant ID__ __ __ Date __ __/__ __/__ __ __ __

Staff initials __ __ __

# Exit interview- Social supporters

*Instructions: Complete this form at disenrollment from the study. Bring a summary of the SMS notifications sent and a calendar to review, if needed, to assist the participant with answering the questions.*

1. complete the following if a social supporter was sent at least one SMS notification, else, skip to question 1b

Did you receive the SMS notification?

Yes

No

Don’t remember

Did you attempt to contact the Wisepill participant?

Yes

No (why?)______________

Don’t remember

Did you provide help to the Wisepill participant?

No (why?)_____

Yes (what kind of help?)

- Money
- Ride to clinic__
- Emotional support_
- Other (specifiy)___________

b.*If no or the participant did not receive all sent SMS notifications, ask:*

Are there instances when you needed to help AA (patient name) but could not?

Yes (please give reasons)…………………………………………………

No

Why do you think you did not receive the SMS notification(s)? (*tick all that apply*)

My phone may have been turned off

My phone may not have had power

I switched to a new phone number

The network may not have been available

I have no idea

Other (specify) _______________________________________________

______________________________________________________

2. Are you aware of any times when the Wisepill participant did not take his/her TB medications for more than 48 hours and you did not receive an SMS notification?

Yes No -->skip to question 3

*If yes,* please explain the circumstances _____________________________________

_____________________________________________________________________

_____________________________________________________________________

3. How did you feel about receiving the SMS notifications?

Very much disliked Disliked Liked Very much liked

4. How is your relationship with the Wisepill participant now compared to how it was when you started the study?

*greatly improved* *slightly improved* *No change* *Slightly declined*

*Greatly declined*

Give reasons to support your answer ____________________________________________

5. Do you think your support/or lack of support to the participant has been helpful and encouraging in any way? *Yes* *No*

Please explain……............................................................................................................................................ (interviewer may probe for a detailed response).

6. What motivated you to help or not to help the wisepill participant?____________________________________________________________________________________________________________________________________________________________________________________________

7. Have you ever cared for someone with TB or on TB medication besides this participant?

*Yes* *No*

8. How often do you communicate, visit or discuss with the wisepill participant?

*At least daily* *Several times a week* *Once a week* *Once in 2 weeks* *Once a month* *Over a month* *Never*

9a. How interested would you be in continuing to receive SMS notifications for the Wisepill participant?

Not interested at all Somewhat interested Very interested

9b. Which of the following describes you (tick as many as applicable)?

*For those who received daily SMS*

I preferred daily SMS notifications to notifications that were coming after AA (patient name) has missed medication

I preferred SMS notifications that were coming after AA (patient name) missed medication than daily SMS notifications.

Though I received daily SMS notifications, I would have preferred to receive weekly notifications

Please give reasons for your answer(s)…………………………………………………………

*For those who received weekly SMS*

I preferred SMS notifications that were coming after AA (patient name) missed medication than weekly SMS notifications.

I preferred weekly SMS notifications to notifications that were coming after AA (patient name) missed medication

Though I received weekly SMS notifications, I would have preferred to receive daily notifications

Please give reasons for your answer………………………………………………………….

9c.- Which of the following describes the type of SMS notifications you preferred to receive

The default SMS notification (i.e. “T*his is your reminder”*)

Personalized SMS (i.e. reminders formulated by patients e.g. “take care”)

10. Is there anything else you would like to tell me about the study?

*You finished!*

*✿****Thank you for your help****✿*
