# Supplementary material for: The feasibility, acceptability, and preliminary impact of real-time monitors and SMS on tuberculosis medication adherence in southwestern Uganda: Findings from a mixed methods pilot randomized controlled trial
Source: PLOS Glob Public Health. 2023 Dec 5;3(12):e0001813. doi: 10.1371/journal.pgph.0001813 (PMC10697590; doi:10.1371/journal.pgph.0001813)
Supplement: Attachment — Submitted filename: PGPH-D-23-00372_Feedback to reviewers_12th May.docx [file pgph.0001813.s006.docx]

PGPH-D-23-00372
The Feasibility, Acceptability, and Preliminary Impact of Real-Time Monitors and SMS on Tuberculosis Medication Adherence: Findings from a Pilot Randomized Controlled Trial
PLOS Global Public Health

Dear Dr. Musiimenta,

Thank you for submitting your manuscript to PLOS Global Public Health. After careful consideration, we feel that it has merit but does not fully meet PLOS Global Public Health’s publication criteria as it currently stands. Therefore, we invite you to submit a revised version of the manuscript that addresses the points raised during the review process.

Please submit your revised manuscript by 08 May 2023. If you will need more time than this to complete your revisions, please reply to this message or contact the journal office at [globalpubhealth@plos.org](mailto:globalpubhealth@plos.org). When you're ready to submit your revision, log on to <https://www.editorialmanager.com/pgph/> and select the 'Submissions Needing Revision' folder to locate your manuscript file.

Please include the following items when submitting your revised manuscript:

- A rebuttal letter that responds to each point raised by the editor and reviewer(s). You should upload this letter as a separate file labeled 'Response to Reviewers'.
- A marked-up copy of your manuscript that highlights changes made to the original version. You should upload this as a separate file labeled 'Revised Manuscript with Track Changes'.
- An unmarked version of your revised paper without tracked changes. You should upload this as a separate file labeled 'Manuscript'.

Guidelines for resubmitting your figure files are available below the reviewer comments at the end of this letter.

We look forward to receiving your revised manuscript.

Kind regards,

Dr. Lucy Chimoyi, PhD
Academic Editor
PLOS Global Public Health

Journal Requirements:

1. Please provide separate figure files in .tif or .eps format only and remove any figures embedded in your manuscript file. Please also ensure that all files are under our size limit of 10MB.

For more information about figure files please see our guidelines:

<https://journals.plos.org/globalpublichealth/s/figures> 
<https://journals.plos.org/globalpublichealth/s/figures#loc-file-requirement>

2. Please amend your detailed Financial Disclosure statement. This is published with the article. It must therefore be completed in full sentences and contain the exact wording you wish to be published.

a. State the initials, alongside each funding source, of each author to receive each grant.

Response: We appreciate the Editor for this comment, we have now revised the financial disclosure statement as guided to read as below

**Funding**: This work was supported by a grant from the Fogarty International Center (FIC) of the National Institutes of Health (NIH) (K43TW010388) to AM. The contents of this article are solely the responsibility of the authors and do not necessarily represent the official views of the Fogarty International Center of the National Institutes of Health. The funders had no role in study design, data collection and analysis, decision to publish, or preparation of the manuscript. JEH is supported by the NIH grant (K24MH114732). ECA is supported by the FIC of the NIH (K43TWO11004). Authors AM, AMT and WT received salaries from the K43TW010388 grant funded by the FIC/NIH

b. State what role the funders took in the study. If the funders had no role in your study, please state: “The funders had no role in study design, data collection and analysis, decision to publish, or preparation of the manuscript.”

***Response:*** *In the* financial disclosure statement*, we have now incorporated the following statement “The funders had no role in study design, data collection and analysis, decision to publish, or preparation of the manuscript.”*

c. If any authors received a salary from any of your funders, please state which authors and which funders. If you did not receive any funding for this study, please simply state: “The authors received no specific funding for this work.”

**Response:** The following statement has now been added to show the authors who received salaries from the grant that funded the work.

“Authors AM, ATM and WT received salaries from the K43TW010388 grant funded by the FIC/NIH”

3. We have noticed that you have uploaded Supporting Information files, but you have not included a list of legends. Please add a full list of legends for your Supporting Information files after the references list.

**Response:** We have now included the full list if legends for the supporting information files after the reference list

4. In the online submission form, you indicated that "The datasets analysed during the current study are available from the corresponding author on reasonable request". All PLOS journals now require all data underlying the findings described in their manuscript to be freely available to other researchers, either 1. In a public repository, 2. Within the manuscript itself, or 3. Uploaded as supplementary information.
This policy applies to all data except where public deposition would breach compliance with the protocol approved by your research ethics board. If your data cannot be made publicly available for ethical or legal reasons (e.g., public availability would compromise patient privacy), please explain your reasons by return email and your exemption request will be escalated to the editor for approval. Your exemption request will be handled independently and will not hold up the peer review process, but will need to be resolved should your manuscript be accepted for publication. One of the Editorial team will then be in touch if there are any issues.

**Response:** We appreciate the Editor for this comment, data from this study contain sensitive patient information (e.g. HIV/TB status, patients’ medication taking histories, telephone contacts) which may compromise the safety of the patients’ information. Also, sharing this data breach the compliance with the protocol approved our research ethics board. We have now revised the section as guided to read as follows.

Data from this study contain sensitive patient information. Also, sharing this data breach the compliance with the protocol approved our research ethics board. However, de-identified individual data that supports the results will be shared following publication provided the investigator who proposes to use the data has approval from an Institutional Review Board (IRB), Independent Ethics Committee (IEC), or Research Ethics Committee (REC), as applicable, and executes a data use/sharing agreement with Mbarara University of Science and Technology (MUST). Researchers may apply for data access by contacting the corresponding author or the MUST Research Ethics Committee at [sec@rec.must.ac.ug](mailto:sec@rec.must.ac.ug).

Additional Editor Comments (if provided):

1.The qualitative data collection tools were developed using the UTAUT. I am assuming that the analysis and presentation of results followed this theory. However, this is not clear from Table 4. Include the theory used as part of the caption.

**Response:** As indicated in the manuscript under “Data Analysis” section

Qualitative data was analyzed using content analysis to generate categories summarizing participants’ experiences of using the technologies, focusing on perceived usefulness and challenging issues counting for effort, social influences and facilitators. Although UTAUT guided the development of data collection tools, the analysis did not exactly follow the model as this would restrict the possibility of discovering themes that not included in the UTAUT model.

2. It is advisable for authors to include in their title that this manuscript followed a mixed-methods approach.

**Response:** We appreciate this comments, we have now revised the title as follows:

**The Feasibility, Acceptability, and Preliminary Impact of Real-Time Monitors and SMS on Tuberculosis Medication Adherence: Findings from a Mixed Methods Pilot Randomized Controlled Trial**

3. A sub-section on outcome measures in the methods section should be included and all outcome measures defined (feasibility, acceptability and adherence).

**Response:** We appreciate this comment. We have now added the outcome measures section in the methods section on page 8. This section reads:

The primary outcome was percent adherence, number of late doses, number of missed doses, treatment completion rate, treatment success rate ascertained by the real time monitor. Secondary outcomes were feasibility which was verified by the technical function of the intervention including the number of SMS sent as planned, number of SMS not sent as planned, number of SMS sent unnecessarily, data loss by the real-time monitor, monitor malfunctioning, taking medication from other sources, and opening the device without taking medication. Acceptability of the intervention verified by the preference of SMS type and frequency, ease of use of the real-time monitor, possibility of unintended TB status disclosure.

4.From this work, do you think that poor network coverage is a problem or a limitation? This has been mentioned in the results, but authors have not discussed its implication. Is it something that implementers need to be concerned about?

**Response:** In the discussion section, we noted that “Notably, cellphone network is rapidly expanding in Uganda (36), and there is widespread cellphone adoption including among PLTB (37), suggesting these technical challenges may improve with time”.

We have now added the following sentence

“With 2G networks reaching nearly the entire population in Uganda, and with mobile broadband networks (3G/4G) covering more than 80% of the country (39), poor network is not a significant challenge.”

5. Thank you for including the CONSORT checklist, also include the COREQ checklist

**Response:** You are welcome. We have now included the COREQ checklist as advised.

6. Was saturation determined during analysis of the qualitative data?

**Response:** Typical of qualitative methods, we co-currently carried interviews and analysis. Yes the saturation was determined at the 30^th^ participant during analysis and this has now been included under data collection section as follows:

…authors WT and ATM (who are trained in qualitative research and research ethics) conducted semi-structured, face to face in-depth interviews with PLTB in a private space at a research office near the MRRH until reaching thematic saturation at the 30^th^ participant.

[Note: HTML markup is below. Please do not edit.]

Reviewers' comments:

Reviewer's Responses to Questions

**Comments to the Author**

1. Does this manuscript meet PLOS Global Public Health’s [publication criteria](https://journals.plos.org/globalpublichealth/s/criteria-for-publication)? Is the manuscript technically sound, and do the data support the conclusions? The manuscript must describe methodologically and ethically rigorous research with conclusions that are appropriately drawn based on the data presented.

Reviewer #1: Yes

Reviewer #2: Yes

Reviewer #3: Yes

2. Has the statistical analysis been performed appropriately and rigorously?

Reviewer #1: Yes

Reviewer #2: Yes

Reviewer #3: Yes

3. Have the authors made all data underlying the findings in their manuscript fully available (please refer to the Data Availability Statement at the start of the manuscript PDF file)?

The [PLOS Data policy](https://journals.plos.org/globalpublichealth/s/data-availability) requires authors to make all data underlying the findings described in their manuscript fully available without restriction, with rare exception. The data should be provided as part of the manuscript or its supporting information, or deposited to a public repository. For example, in addition to summary statistics, the data points behind means, medians and variance measures should be available. If there are restrictions on publicly sharing data—e.g. participant privacy or use of data from a third party—those must be specified.

Reviewer #1: No

Reviewer #2: No

Reviewer #3: No

4. Is the manuscript presented in an intelligible fashion and written in standard English?

PLOS Global Public Health does not copyedit accepted manuscripts, so the language in submitted articles must be clear, correct, and unambiguous. Any typographical or grammatical errors should be corrected at revision, so please note any specific errors here.

Reviewer #1: Yes

Reviewer #2: Yes

Reviewer #3: Yes

5. Review Comments to the Author

Please use the space provided to explain your answers to the questions above. You may also include additional comments for the author, including concerns about dual publication, research ethics, or publication ethics. (Please upload your review as an attachment if it exceeds 20,000 characters)

Reviewer #1: This article reports the feasibility and acceptability of a piloted TB adherence support strategy that combines a medication reminder box and SMS reminders to both patients and supporters. The study is a valuable addition to the TB digital adherence technology literature, but there are several aspects where I believe that there is incomplete reporting or a lack of transparency/clarity in the methods and results.

1) The second paragraph of the introduction refers to DOTS, which I find confusing because of the way this acronym has been used by WHO. The authors are discussing the practice of directly observed therapy (commonly abbreviated DOT), and I agree with everything they say about its shortcomings. However, the authors use the abbreviation DOTS (with the terminal S), which is generally used to refer to the old WHO DOTS Strategy. This strategy was included much more than DOT. It had five components - government commitment, a reliance on smear microscopy, directly observed short-course treatment, uninterrupted drug supply, and standardized reporting; even the third component (which corresponds to the phrase “directly observed therapy – short course” mentioned by the authors) encompassed not only the practice of DOT but the specific regimens that were recommended at the time. I think the authors should make it clear that they are talking about the practice of DOT, not the DOTS Strategy. This confusion could be resolved by simply defining the phrase “directly observed therapy (DOT)” in the first sentence of the paragraph, and using the abbreviation DOT rather than DOTS throughout.

**Response**: We would like to thank the reviewer for guiding on this, and we apologize for this confusion. We have now replaced DOTS with DOT

2) In the statistical analysis section, I am a little confused by the use of the term “censoring.” My understanding of the term censoring in statistics is that it is used (usually for survival analysis) when partial information is known about the outcome, specifically that the event of interest has not yet occurred by a certain point, but that it will happen in the future. If I am understanding the analysis correctly, the authors did not use a survival analysis – I believe they used a linear regression with adherence as a continuous outcome. There are various periods of time that are excluded from the adherence calculation (e.g. time after death), but I do not think that this is censoring because the information about the outcome is not in doubt (participants could not have been adherent after death), and the regression model is not treating it as such. Maybe it would be clearer to just say that these days were excluded from the adherence calculation (i.e. excluded from both numerator and denominator)?

**Response:** As advised, we have changed censoring to “excluded” wherever applicable.

3) A definition of unnecessary SMS (reported in Table 2) and how it was determined should be included in the methods.

**Response:** We thank you for this comment. We have now included the following description on page 17 under the section for “feasibility and acceptability”— “SMS reminders and notifications”

Only 7% of SMS were sent unnecessarily due to technical issues (such as poor network coverage). Most SMS reminders sent unnecessarily occurred when the electronic adherence monitor was opened to take medication, but there was delay or absence of the corresponding signal due technical issues (such as poor network coverage).

4) I suggest that the authors include the questionnaire as a supplementary file. It is unclear how some of the information in Tables 2 and 3 and the text was elicited (specifically the sections “SMS reminders and notifications”, “SMS notifications to social supporters”, and “real-time adherence monitor”). For example, how were patients asked about their preference for daily/weekly versus triggered SMS reminders given that they presumably received both? Do the responses mean that they would have wanted *only* the triggered reminders? How was ease of use assessed – a binary answer of “easy” or “difficult” or a Likert scale? Was fear of disclosure specifically asked about, or did 5% report this unintended consequence in the open-ended part of the questionnaire? What other forms of support could have been reported by treatment supporters.

**Response:** We have now attached the questionnaire as a supplementary file

5) Tables 2 and 3 mix indicators that were collected via the device, as well as things that I assume the authors could only know by asking the patient, and things that may have been recorded through the study teams’ field notes. This makes it a little confusing to evaluate, and it makes me wonder why these particular indicators are being reported in the tables. One way to improve transparency would be to add a column or footnotes to the table indicating the source of each data element. Another would be to include the questionnaire as supplementary information as noted above.

**Response:** Thank you. We have now added a footnote indicating “preference of SMS type/frequency” was reported by participants.

6) How many subjects participated in interviews? Did the interviewees know that the interviewers were representatives of the study that provided the monitors (raising the possibility of social acceptability bias in responses)? (I am not going to go down the COREQ checklist and raise every point that is not included in the methods, but I think that these two points are particularly important for judging validity).

**Response:** We appreciate the comment. 30 participants were interviewed. All participants were briefed on the purpose of the study. We have now included social desirability as potential limitation of the study.

“Since participants self-reported their perceptions about the intervention, qualitative results (and some of the self-reported quantitative aspects) could be vulnerable to social desirability bias”.

We have also attached the COREQ checklist that has the full details.

7) In the qualitative interviews, were there minority opinions of participants who contradicted the general themes included under “perceived usefulness”? For instance, was there anyone who said that the medication reminder was annoying and that they would have remembered to take their meds anyway since they are used to taking daily ART? The qualitative results reported are rather scanty, and all but one of the themes reported about the technology itself are positive. This raises the concern that negative or ambivalent opinions are not being reported to make a case for acceptability.

**Response:** We report two potentially ambivalent opinions in Table 4 of qualitative results. These are possibility of unintended TB status disclosure and inability to address structural barriers to medication adherence.

8) The second paragraph of the discussion is confusing in that the first sentence claims, “the use of social support notifications to social supporters was feasible,” while the third sentence says “the feasibility of SMS notifications to social supporters in our study was limited.” The following sentences then talk about the need to improve feasibility. I think that the authors are either mixing the discussion of feasibility of two different things, or, starting with the third sentence, they are actually making a different point that is not about feasibility. The first sentence is clearly talking about the feasibility of delivering SMS notifications to social supporters. The remaining sentences are discussing whether these notifications are likely to have an impact given the limited ability of social supporters to overcome socioeconomic barriers. To me, this is a different point that is not about feasibility. However, the authors may be conceptualizing the point as a broader discussion about the feasibility of a hypothetical social support intervention that could utilize the SMS messages – not the actual intervention they piloted. I am fine with either conceptualization, but it has to be clear *what* is feasible or not.

Response: Thank you for your comment. We have now revised the sentence about the limited feasibility to read as follows:

“However, the impact of SMS notifications could be constrained by the reported lack of financial resources to support TB patients.

Minor:
Line 342 reports that “participants who had disclosed their status” had lower adherence. What statis was being disclosed and to whom?

**Response:** We have now clarified the sentence to read as follows:

… participants who had disclosed their status (n=17; 83%) to anyone outside of their care provider had 8.3% (95% CI -18.8, 2.1, P=0.12)

Line 443 – I think the authors mean discreet, not discrete

**Response:** Thank you. We have now revised it to discreet as advised.

**Reviewer #2: Thank you authors for this informative manuscript. Kindly find herewith some suggestions and questions that would help improve this work.**

Line 167 - 168 ? -- What criteria was used by the Wisepill to determine acceptability and feasibility?

**Response:** We appreciate the reviewer for this comment. Under “outcome measures” section on page 9, we now included the following information:

Secondary outcomes were feasibility which was verified by the technical function of the intervention including the number of SMS sent as planned, number of SMS not sent as planned, number of SMS sent unnecessarily, data loss by the real-time monitor, monitor malfunctioning, taking medication from other sources, and opening the device without taking medication. Acceptability of the intervention verified by the preference of SMS type and frequency, ease of use of the real-time monitor, possibility of unintended TB status disclosure.

Line 263 "Overall, a total of 39 social supporters completed the study"
line 266 "Of the 42 social supporters"
Line 295 "All social supporters (n = 39; 100%)
Please clarify the correct number of social supporters analyzed.

**Response:** In the description following the CONSORT diagram, we state that:

“Of the 45 social supporters, three were disenrolled all from the weekly SMS arm after 3.5, 6.5, and 12.2 weeks respectively and their data was excluded for analysis because of disenrollment of their corresponding PLTB. Data from the three social supporters whose PLTB died (from the daily SMS arm) before completing the study were included for analysis. Overall, a total of 39 social supporters completed the study”.

line 259 - "..totaling to 63 PLTB"
Line 269 Table 1 - baseline demographics characteristics shows (N=63)
Line 307 -- "All PLTB completing the study (n=58; 100%)"
Please clarify the correct number of PLTB analyzed.

**Response**: We thank you for this comment. In the description following the CONSORT diagram, we now clarify by adding the following description:

However, due to the three patients who died from the daily SMS arm and one who died and the other one who was lost to follow up (due to unreachable contact in the control arm), we only had 58 PLTB at study exit; 19 PLTB in the daily SMS arm, 20 in the weekly SMS arm, and 19 in the control arm.

Line 315; Table 3, Reason(s) (n = 10)
However, the corresponding total number of the participants in the three reasons is greater than 10 i.e. (7+3+8 = 18)

**Response:** We apologize for this confusion. We have now revised the table accordingly and removed unnecessary information that was causing the anomaly.

Deeper analysis based on stratifying the TB treatment phases; intensive phase (2 months) and continuation phase (4-6 months) based on the frequency of reminders could further inform the non-adherence

**Response:** We appreciate the reviewer’s suggestion and have since conducted an analysis looking at adherence by phase and study arm. Having controlled for study arm, we found no difference in adherence between the two phases. Likewise, the difference between either intervention arms and the control arm did not differ over the two phases. We have revised the methods and results as follows:

Methods

First, we built univariable models considering all potential confounders, which included age, gender, marital status, type of residence, disclosure status, severe food insecurity, alcohol use, stigma, social support, depression, HIV status, social economic status, study phase (less or equal to 2 months of treatment vs more than 2 months of treatment)

Results

The median adherence over the 6-month study period was 96.1% (84.8, 98.0), 92.5% (80.6, 96.3), and 92.2% (56.3, 97.8) respectively in the daily SMS, weekly SMS, and control arms and was similar, within study arm, over the two study phases.

During the qualitative data analysis, did any patient raise the issue of SMS reminders fatigue due to the daily frequency of incoming messages as reported in other studies? JAMA Intern Med. 2016;176(3):340-349. doi:10.1001/jamainternmed.2015.7667

**Response:** Participants in our study didn’t report any technological fatigue.

Overall grammatical corrections to improve readability

**Response:** We thank you for this comment. We have now corrected grammatical errors throughout the manuscript.

Reviewer #3: Congratulations to this team for a fantastic study and a comprehensive and interesting evaluation of real-time adherence monitoring. The combination of qualitative data provides a nice overview of the benefits and potential drawbacks of this approach. Below I list a number of issues, nearly all minor, that should be clarified.

Author affiliations – no one is assigned 5 or 6.

**Response**: First, we appreciate the reviewer’s encouraging comments.

We apologize for the oversight; the affiliation numbering has now been revised.

Abstract: please specify primary outcomes. Was adherence a primary outcome?

**Response**: We appreciate this comment. We have now included the following sentence to specify the primary outcomes in the abstract.

“The primary outcome was percent adherence as ascertained by the real time monitor”

Abstract: Please add confidence intervals around adherence percentages – remove reference to “may have improved adherence” as there is little evidence cited in the abstract for this. Alternatively cite the adjusted adherence findings with confidence intervals and could leave this in. See my note at the end regarding statistical significance.

Response: Thank you for this suggestion. We have now included the confidence intervals as indicated below:

“Median adherence (IQR) in the daily SMS, weekly SMS, and control arms was 96.1% (84.8, 98.0), 92.5% (80.6, 96.3), and 92.2% (56.3, 97.8), respectively”.

Throughout the paper there seems to be a conflation of DOTS (the broader 5 tenet strategy) and DOT (directly observed treatment), which is an element of DOTS. To my read, DOTS is used in places where DOT, but not DOTS, would be appropriate.

**Response:** We apologize for this confusion. We have now revised this to document DOT.

Line 93 – minor typo “bn”

**Response:** Thank you. This has now been deleted.

Page 5 refers to formative findings from the current study and then a formative study. Which is correct? Or are these references to two different studies?

**Response:** We apologize for unnecessarily repeating the phrase “formative study”. We have now deleted this repetition. To clarify, we did a formative study at the beginning of the current study in which we published two articles (Musiimenta et al 2019 and Musiimenta et al 2020) cited in the text on page 5.

Line 135 p6: Change “cater for” to “account for”

**Response:** This has now been changed as guided

P6, line 138 – what does it mean to recruit them at random? Was this a convenience sample? If not, explain the systematic for recruitment

**Response:** We have now revised this to read as below “we randomly recruited PLTB if they met the following.” this randomization is clearly discussed under the study procedures section, line 179-183 as shown below

*PLTB participants were followed for six months. A random number generator (random allocation was generated by the study coordinator) and the CONSORT diagram (figure 1 below) were used to determine study arm assignments by the study team. After screening and consenting, all PLTB participants received a real-time adherence monitoring device and were randomized 1:1:1 as follows:..*

Line 143 – Explain how access to cellular network was assessed.

**Response:** Thank you for this comment, we have now revised the sentence to read as follows:

… cellular reception was assessed by the research assistants who escorted participants in their homes to check the signal of the networks supported by the technology used in this study either MTN or Airtel.

Not clear how purposive sampling fits in to the design. To whom does this apply? People with TB? Supporters? Both?

**Response:** We appreciate this comment. As indicated in the manuscript, we used purposive sampling to achieve relatively balanced representation by gender, given the potential gender differences in TB medication adherence (25).

Could a patient participate if they did not have a supporter?

**Response:** Per the eligibility criteria, patients were only recruited if they had a social supporter

How often and at what frequency were SMS reminders sent to supporters? Was it the same as for the PLTB?

**Response:** The SMS reminders sent to social supporters were sent at the same time as the PLTB

Primary outcomes are defined as adherence but as the authors note, there was insufficient power for these outcomes. Were these really the primary outcomes or were the primary outcomes feasibility and acceptability?

**Response:** The primary outcome was percent adherence as ascertained by the real time monitor, while secondary outcomes were feasibility and acceptability.

Line 193 – what do the authors mean by non-responsiveness?

**Response:** We have now clarified that non-responsiveness means not taking medication as expected.

When was the data censored in patients with LTFU? At the end of the two months without treatment or the beginning of those first two months?

**Response:** Data were censored at the onset of the missing data that qualified the participant as LTFU. We have changed the text accordingly specifying that for the LTFU, data were excluded from the onset of the missing data.

Line 223 – perhaps “secondary data analysis” instead of “dataset”?

**Response:** Thank you. We have now replaced “dataset” with “analysis”

Why were HIV and social support, versus other variables, considered a priori confounders? Especially in the context of a trial?

**Response:** We apologize for the oversight and since removed this information. We only controlled for study site and have updated the text to this effect. The text reads as follows; …we considered those confounders with a univariable p value <0.2, also controlling for study arm.

Line 263 – 39 of 45 completed the study but the text only explains that 3 of the 45 were excluded. What happened to the other 3?

**Response:** In the description following the CONSORT diagram, we state that:

“Of the 45 social supporters, three were disenrolled all from the weekly SMS arm after 3.5, 6.5, and 12.2 weeks respectively and their data was excluded for analysis because of disenrollment of their corresponding PLTB. Data from the three social supporters whose PLTB died (from the daily SMS arm) before completing the study were included for analysis. Overall, a total of 39 social supporters completed the study”.

Nearly 25% of messages were not received – this demonstrates feasibility and room for improvement; worth highlighting this in the discussion

**Response:** Actually, as indicated in Table 2, 504/3,267 (15%) of SMS reminders were either not sent or delayed. We have now emphasized this feasibility in the discussion.

Table 1 is very nice.

**Response:** Thank you.

Table 2 is also nice, but it would be useful to have the percentage of daily, weekly and triggered messages received rather than the percentage of messages received that were daily, weekly, triggered. Same with messages not sent.

**Response:** We appreciate this suggestion. We have now revised the tables as suggested.

In tables – use Severely Food Insecure or Severe Food Insecurity

**Response:** This has now been changed

Possible to differentiate between messages not sent and those that were delayed – it seems like this is an important distinction.

**Response:** We now indicate messages not delivered, as well as messages sent unnecessarily (e.g. due to delays)

Could provide more info on unnecessary messages

**Response:** We thank you for this comment. We have now included the following description on page 17 under the section for “feasibility and acceptability”— “SMS reminders and notifications”

Only 7% of SMS were sent unnecessarily due to technical issues (such as poor network coverage). Most SMS reminders sent unnecessarily occurred when the electronic adherence monitor was opened to take medication, but there was delay or absence of the corresponding signal due technical issues (such as poor network coverage).

Lines 297-299 is difficult to understand – could be reworded for clarity

**Response:** We have now revised this to improve clarity as follows:

“The assistance that involved some form of financial spending (such as providing money for transport to the clinic, buying food) was provided least often (n=20; 51%) Non-monetary support such as emotional support was the most common support provided (n=38; 97%)”.

Lines 302 – 305 could be further explained.

**Response:** We had now added some explanation.

Table 3 - Opening the monitor without taking pills is in the wrong place I think

**Response:** We apologize for this mistake. We have now deleted it.

Qualitative table: Lack of money – is this the right title for this theme? It doesn’t relate to the technology. A more suitable title might be “inability to address structural causes of non-adherence” or something along those lines. Adherence monitoring can overcome forgetfulness and perhaps mitigate others, but is unlikely to triumph over extreme poverty.

**Response:** We appreciate this comment, and thank the review for suggesting the most appropriate term for theme. We have now revised the theme to “inability to address structural barriers to non-adherence”

Lines 333/3 – please add labels to the numbers in parentheses. Are these confidence intervals? Ranges?

**Response:** We have now put labels to the numbers in the parentheses. These are the Interquartile ranges.

There is an adjusted analysis in lines 335/6. The results of an equivalent, unadjusted analysis should precede the adjusted analysis.

**Response**: We apologize for the oversight and since removed this information. We only controlled for study site and have updated the text to this effect. The text reads as follows; “…we considered those confounders with a univariable p value <0.2”, also controlling for study arm.

Disclosure is adjusted for but not included in the table 1.

**Response**. We have included information about disclosure in Table 1 whereby 52 (83%) of patients reported to have ever disclosed their TB status to anyone other than healthcare provider.

Suggest removing the findings related to gender, and food insecurity because the analyses are underpowered, these findings are outside the objective of this analysis and you did not look at specific confounders for these variables so these estimates cannot be interpreted in the same way as your main exposure variables (study arm).

**Response**: As suggested, we have omitted these analyses and now only present the main exposure results.

Throughout the paper, suggest de-emphasizing lack of statistical significance. You weren’t adequately powered for statistical significance. Instead, would highlight preliminary evidence for a potential clinically meaningful effect that should be evaluated in a larger trial

**Response:** We appreciate this comments. We have now de-emphasized lack of statistical significance throughout the paper.

Please add reference to ASCENT trial or [clinicaltrial.gov](http://clinicaltrial.gov) number

**Response:** We have now included the ClinicalTrials.gov number, [NCT02574455](http://clinicaltrials.gov/show/NCT02574455)

369 – not sure the adherence monitoring via hair/urine really fits in here. I don’t think you are suggesting that these should be used programmatically. What are you are suggesting? Consider removing.

**Response:** Thank you. Hair/urine analysis has been removed.

381 (devise, not device); also “farming” versus “faming”
**Response:** We appreciate this comment have now revised the words as advised.

Worth one final close proofread for typographical edits

6. PLOS authors have the option to publish the peer review history of their article ([what does this mean?](https://journals.plos.org/globalpublichealth/s/editorial-and-peer-review-process#loc-peer-review-history)). If published, this will include your full peer review and any attached files.


**Do you want your identity to be public for this peer review?** If you choose “no”, your identity will remain anonymous but your review may still be made public.


For information about this choice, including consent withdrawal, please see our [Privacy Policy](https://www.plos.org/privacy-policy).

Reviewer #1: No

Reviewer #2: No

Reviewer #3: No

[NOTE: If reviewer comments were submitted as an attachment file, they will be attached to this email and accessible via the submission site. Please log into your account, locate the manuscript record, and check for the action link "View Attachments". If this link does not appear, there are no attachment files.]

While revising your submission, please upload your figure files to the Preflight Analysis and Conversion Engine (PACE) digital diagnostic tool, <https://pacev2.apexcovantage.com/>. PACE helps ensure that figures meet PLOS requirements. To use PACE, you must first register as a user. Registration is free. Then, login and navigate to the UPLOAD tab, where you will find detailed instructions on how to use the tool. If you encounter any issues or have any questions when using PACE, please email PLOS at [figures@plos.org](mailto:figures@plos.org). Please note that Supporting Information files do not need this step.
