# Supplementary material for: The feasibility, acceptability, and preliminary impact of real-time monitors and SMS on tuberculosis medication adherence in southwestern Uganda: Findings from a mixed methods pilot randomized controlled trial
Source: PLOS Glob Public Health. 2023 Dec 5;3(12):e0001813. doi: 10.1371/journal.pgph.0001813 (PMC10697590; doi:10.1371/journal.pgph.0001813)
Supplement: Attachment — Submitted filename: PGPH-D-23-00372_Response to Reviewers.docx [file pgph.0001813.s007.docx]

Top of Form

**View Letter**

Bottom of Form

| Date: | Jun 20 2023 08:39PM |
| --- | --- |
| To: | "Angella Musiimenta" amusiimenta@must.ac.ug |
| cc: | megan.coffee@nyulangone.org, megan.coffee@gmail.com |
| From: | "PLOS Global Public Health" globalpubhealth@plos.org |
| Subject: | PLOS Global Public Health Decision: Revision required [PGPH-D-23-00372R1] |

PGPH-D-23-00372R1
The Feasibility, Acceptability, and Preliminary Impact of Real-Time Monitors and SMS on Tuberculosis Medication Adherence: Findings from a Mixed Methods Pilot Randomized Controlled Trial
PLOS Global Public Health

Dear Dr. Musiimenta

Thank you for submitting your manuscript to PLOS Global Public Health. After careful consideration, we feel that it has merit but does not fully meet PLOS Global Public Health’s publication criteria as it currently stands. Therefore, we invite you to submit a revised version of the manuscript that addresses the points raised during the review process.

There has been substantial improvement of the paper and we appreciate this work. The paper is much closer to publication. Please see the comments and edits outlined by Reviewer 1. These comments would best guide the changes that are needed.

Please submit your revised manuscript by August 1. If you will need more time than this to complete your revisions, please reply to this message or contact the journal office at globalpubhealth@plos.org. When you're ready to submit your revision, log on to https://www.editorialmanager.com/pgph/ and select the 'Submissions Needing Revision' folder to locate your manuscript file.

Please include the following items when submitting your revised manuscript:

- A rebuttal letter that responds to each point raised by the editor and reviewer(s). You should upload this letter as a separate file labeled 'Response to Reviewers'.
- A marked-up copy of your manuscript that highlights changes made to the original version. You should upload this as a separate file labeled 'Revised Manuscript with Track Changes'.
- An unmarked version of your revised paper without tracked changes. You should upload this as a separate file labeled 'Manuscript'.

Guidelines for resubmitting your figure files are available below the reviewer comments at the end of this letter.

We look forward to receiving your revised manuscript.

Kind regards,

Megan Coffee, MD, PhD
Academic Editor
PLOS Global Public Health

Journal Requirements:

Additional Editor Comments (if provided):


[Note: HTML markup is below. Please do not edit.]

Reviewers' comments:

Reviewer's Responses to Questions

**Comments to the Author**

1. If the authors have adequately addressed your comments raised in a previous round of review and you feel that this manuscript is now acceptable for publication, you may indicate that here to bypass the “Comments to the Author” section, enter your conflict of interest statement in the “Confidential to Editor” section, and submit your "Accept" recommendation.

Reviewer #1: (No Response)

Reviewer #2: (No Response)

2. Does this manuscript meet PLOS Global Public Health’s [publication criteria](https://journals.plos.org/globalpublichealth/s/criteria-for-publication)? Is the manuscript technically sound, and do the data support the conclusions? The manuscript must describe methodologically and ethically rigorous research with conclusions that are appropriately drawn based on the data presented.

Reviewer #1: Partly

Reviewer #2: Yes

3. Has the statistical analysis been performed appropriately and rigorously?

Reviewer #1: N/A

Reviewer #2: Yes

4. Have the authors made all data underlying the findings in their manuscript fully available (please refer to the Data Availability Statement at the start of the manuscript PDF file)?

The [PLOS Data policy](https://journals.plos.org/globalpublichealth/s/data-availability) requires authors to make all data underlying the findings described in their manuscript fully available without restriction, with rare exception. The data should be provided as part of the manuscript or its supporting information, or deposited to a public repository. For example, in addition to summary statistics, the data points behind means, medians and variance measures should be available. If there are restrictions on publicly sharing data—e.g. participant privacy or use of data from a third party—those must be specified.

Reviewer #1: No

Reviewer #2: Yes

5. Is the manuscript presented in an intelligible fashion and written in standard English?

PLOS Global Public Health does not copyedit accepted manuscripts, so the language in submitted articles must be clear, correct, and unambiguous. Any typographical or grammatical errors should be corrected at revision, so please note any specific errors here.

Reviewer #1: Yes

Reviewer #2: Yes

6. Review Comments to the Author

Please use the space provided to explain your answers to the questions above. You may also include additional comments for the author, including concerns about dual publication, research ethics, or publication ethics. (Please upload your review as an attachment if it exceeds 20,000 characters)

Reviewer #1: Thank you to the authors for their revision. However, most of my concerns from the original review have not been addressed. In addition, now that I am able to see the questionnaires, I have some additional concerns (which I alluded to in the original review but was not able to assess without seeing the data collection forms).

1) My question about participants knowing investigators has not been addressed. Self-report does not alone lead to social desirability bias; knowing that the investigators are the ones who gave them the monitor does. (For example, if the Ministry of Health gave out the monitors and everyone hated them, and the investigators were perceived as being from an organization not aligned with the Ministry of Health, then people might feel perfectly comfortable complaining about them, so it would still be self-report but there would be less risk of social desirability bias).

**Response:** We appreciate this comment from the reviewer. We have now revised the social desirability sentence to incorporate this issue. The revised sentence now reads:

Lastly, since participants self-reported their perceptions about the intervention, qualitative results (and some self-reported quantitative aspects) could be vulnerable to social desirability bias especially since participants knew that they were reporting on the intervention given to them by the same researchers collecting data from them.

2) The COREQ checklist has not been applied properly, and some of the information is still not in the paper. First, the checklist is supposed to reflect the qualitative component of the study, and therefore the information should only pertain to the interview procedures and participants, not the overall study population. Second, the information is supposed to be in the paper, not in the checklist (the checklist just says where to find it in the paper). The purpose of the checklist is to ensure that there is sufficient information in the paper to allow the reader to judge robustness and bias of the qualitative results.

**Response:** We thank the reviewer for this comment. We have now carefully included all the information in the paper as guided.

3) My concerns over the lack of clarity in the tables about where information comes from have to some extent been clarified by attaching the questionnaires. However, seeing the questionnaires has not satisfied my concern about the authors having picked certain indicators to report and not others, and it makes me worry that they have cherry-picked the numbers that make the technology look acceptable. For example:
- Why does Table 2 report the number (%) of people in the weekly reminder group who would have preferred daily reminders but not the number (%) of people in the daily reminder group who would have preferred weekly reminders?

**Response**: We appreciate this comment. None of the participants in the daily SMS reminder group wished to have been receiving the weekly SMS. We did not include this information in the manuscript because we thought it was insignificant. However, we have now included a sentence in Table 2 that reads as follows:

None of the participants who received daily SMS wished they received weekly reminders.

- Table 3 has “Perceived ease of use” reported as a n (%), but as far as I can see in the questionnaire, this question was asked as a Likert item, so it is not obvious what the reported value corresponds to.

**Response**: We have n(%) because all the 58% participants ticked “❑Strongly Agree” to the following question extracted from the questionnaire:

1. It would be easy for me to open the wisepill device to take my TB medication.

❑Strongly Agree ❑Agree ❑Disagree ❑Strongly Disagree.

- Also, the questionnaire actually asked four different questions about ease of use (opening, remember how to get pills, charging, traveling), so it is unclear why the authors only reported the first.

**Response**: We appreciate this comment. We have now included the percentages for remembering how to get pills, and travel. The text in Table 3 now reads:

58 (100%) of PLTB found the real-time monitor easy to open and close, and remember to get pills.

45 (78%) of PLTB found the real-time monitor easy to travel with.

About charging the devices, as indicated in the manuscript, the device battery life is 6 months which is the same as the treatment period for TB, so we anticipated the batteries to be on until the entire treatment period under normal circumstance. However, as indicated in Table 3 participants did not charge any battery, instead, study staff replaced 4 batteries as poor mobile network resulted in repeated attempts to transmit the data which depleted the batteries before the anticipated battery lives.

- The text at line 322 says “As indicated in Table 3, all PLTB completing the study (n=58; 100%) perceived the real-time monitor to be useful in reminding them to take their medications and implying care by healthcare workers.” However, this information is not in Table 3, which only reports ease of use, not usefulness. And I do not know what data support the statement “implying care by healthcare workers.”

**Response**: We appreciate this comment. We have now revised the table to include information about perceived usefulness, and also made appropriate revision to the text as in the manuscript. This text now reads as follows:

As indicated in Table 3, all PLTB (n=58; 100%) reported that using the real-time monitor: a) was useful in their TB medication adherence including being more useful than the pill sackets often used for TB medication; b) helped them take their medication in time/as prescribed; d) positively affected the way they felt about taking medicine; e) and made it easier for PLTB to take their medication.

4) The methods mention that the questionnaires and interview guides were developed based on the Unified Theory of Acceptance and Use of Technology model, which is one of the scientific strengths of this study. Looking at the questionnaire, the “Technology Adoption” section clearly reflects the UTAUT model and I think is the strongest aspect of the questionnaire since it is well grounded in a behavioral theory that can help interrogate the drivers of acceptability and adoption. However, the results from this section of the questionnaire are not really reported, except for isolated questions that have been picked for unclear reasons and collapsed into binary responses. I do not know why the authors chose to ignore this important section of their questionnaire – I realize it is possible that they are saving it for another paper. However, if that is the case, then I would not claim the UTAUT conceptual framework in the methods because the use of the framework is not at all evident in the results.

**Response**: We thank the reviewer for this comment. We have tried to include some of aspects of the model that we had previously left out (e.g. perceived usefulness). Our initial plan was to separate impact assessment from feasibility and acceptability assessment, but we later decided to combine them into one manuscript to produce a richer paper. This however resulted in a voluminous manuscript. We thus decided to leave out some information (especially self-reported information) and mainly focused on reporting information that was objectively captured from the real-time monitor.

5) My concern about specifying data sources for the tables has been inadequately addressed. The Table 2 footnote should specify where all the data come from; I would suggest expanding the footnote to say, “Preference for SMS type/frequency was collected via patient questionnaires; all other data were collected from Wisepill device” (if that is the case). Table 3 should also have a similar footnote indicating where data came from.

**Response:** We appreciate the reviewer for this comment. We have now added the data sources on Tables 2 and 3 as follows:

Table 2: ^*^Preference for SMS type/frequency was collected via patient questionnaires; all other data were collected from Wisepill device.

Table 3: ^*^Data about data loss, device malfunction, and battery changes were collected from Wisepill device. All other data were collected via patient questionnaires.

6) I still do not understand what a message “sent unnecessarily” means. I understand that there were delayed messages when network connectivity failed, but why does that make the message unnecessary?

**Response:** To clarify, we have now revised this to read as messages sent due to a delay or absence of the corresponding signal.

7) I do not understand added phrase at line 330 (“manifested inform of frequent communications)

**Response:** We meant to indicate that participants reported improved relationships with social supporters as a result of being in the study, and that this improvement was seen in their frequent communications. To make it clear, we have now deleted word “manifested”.

Reviewer #2: Thank you authors for this updated manuscript. However, some things are still not that are not clear. The following suggestions and questions might further help in improving this manuscript.

Introduction
The current paper reports on the feasibility, Acceptability, and Preliminary Impact of Real-Time Monitors and SMS on Tuberculosis Medication Adherence: Findings from a Mixed Methods Pilot Randomized Controlled Trial [both the title and introduction line 119] - However, the study number of late doses
Line 35 - Feasibility - What was its definition? How was this it assessed?

**Response:** Thank you for this observation. We define feasibility as the technical function of the intervention including the number of SMS sent as planned, number of SMS not sent as planned, number of SMS sent later due to a delay or absence of the corresponding signal, data loss by the real-time monitor, monitor malfunctioning, taking medication from other sources, and opening the device without taking medication as defined on page 8, lines 174-178.

Response: We have now mentioned how feasibility was accessed by adding a sentence that reads. “Feasibility was mainly verified by the technical function of the intervention at Month 6”

Line 171 - The primary outcome was .. kindly clarify on this, a couple of outcomes are given.

**Response:** We appreciate this comment. We have now revised the sentence on primary outcome to read: “The primary outcome was percent adherence ascertained by the real time monitor.” Line 172.

Impact - How was this it assessed?

**Response:** Impact on adherence was ascertained by the real time monitor. As indicated on page 7, line 161, when opened to take pills, the real-time monitor records a date-and-time stamp; receipt of this signal is interpreted as a proxy for taking medication.

Line 177 - the outcome measure of the acceptability of the intervention is not clear.

**Response:** Acceptability of the intervention was assessed through surveys developed drawing from UTAUT model (Unified Theory of Acceptance and Utilization of Technology). The surveys captured participants’ preference of SMS type and frequency, ease of use of the real-time monitor, possibility of unintended TB status disclosure. Acceptability was also assessed using qualitative in-depth interviews whose guides were also formulated drawing from the UTAUT model.

Line 40: "Among 66 participants " -- Update this to reflect 63 PLTB as indicated on line 269, line 277 and table 1 (line 281)

Response: Thank you. We apologize for this confusion. This has now been revised to reflect 63 rather than 66.

7. PLOS authors have the option to publish the peer review history of their article ([what does this mean?](https://journals.plos.org/globalpublichealth/s/editorial-and-peer-review-process#loc-peer-review-history)). If published, this will include your full peer review and any attached files.


**Do you want your identity to be public for this peer review?** If you choose “no”, your identity will remain anonymous but your review may still be made public.


For information about this choice, including consent withdrawal, please see our [Privacy Policy](https://www.plos.org/privacy-policy).

Reviewer #1: No

Reviewer #2: No

[NOTE: If reviewer comments were submitted as an attachment file, they will be attached to this email and accessible via the submission site. Please log into your account, locate the manuscript record, and check for the action link "View Attachments". If this link does not appear, there are no attachment files.]

While revising your submission, please upload your figure files to the Preflight Analysis and Conversion Engine (PACE) digital diagnostic tool, https://pacev2.apexcovantage.com/. PACE helps ensure that figures meet PLOS requirements. To use PACE, you must first register as a user. Registration is free. Then, login and navigate to the UPLOAD tab, where you will find detailed instructions on how to use the tool. If you encounter any issues or have any questions when using PACE, please email PLOS at figures@plos.org. Please note that Supporting Information files do not need this step.

*In compliance with data protection regulations, you may request that we remove your personal registration details at any time.* [*(Remove my information/details)*](https://www.editorialmanager.com/pgph/login.asp?a=r)*. Please contact the publication office if you have any questions.*

Top of Form

Bottom of Form
