# Supplementary material for: The feasibility, acceptability, and preliminary impact of real-time monitors and SMS on tuberculosis medication adherence in southwestern Uganda: Findings from a mixed methods pilot randomized controlled trial
Source: PLOS Glob Public Health. 2023 Dec 5;3(12):e0001813. doi: 10.1371/journal.pgph.0001813 (PMC10697590; doi:10.1371/journal.pgph.0001813)
Supplement: Attachment — Submitted filename: PGPH-D-23-00372-R3 Response.docx [file pgph.0001813.s008.docx]

PGPH-D-23-00372R2
The Feasibility, Acceptability, and Preliminary Impact of Real-Time Monitors and SMS on Tuberculosis Medication Adherence: Findings from a Mixed Methods Pilot Randomized Controlled Trial
PLOS Global Public Health

Dear Dr. Muslimenta: 

Thank you for submitting your manuscript to PLOS Global Public Health. After careful consideration, we feel that the paper is almost ready for publication and has merit but does not fully meet PLOS Global Public Health’s publication criteria as it currently stands. Therefore, we invite you to submit a revised version of the manuscript that addresses the points raised during the review process.

The paper is close to ready for publication. I do not want to slow down publication but wanted to give you time to incorporate the helpful advice shared by Reviewers 4 and 5. If you are able to share the data with publication that would also be helpful.

Please submit your revised manuscript by October 6, if you are ready. You can take more time if needed, but I just wanted to stress that the paper is almost ready for publication. If you will need more time than this to complete your revisions, please reply to this message or contact the journal office at [globalpubhealth@plos.org](mailto:globalpubhealth@plos.org). When you're ready to submit your revision, log on to <https://www.editorialmanager.com/pgph/> and select the 'Submissions Needing Revision' folder to locate your manuscript file.

Please include the following items when submitting your revised manuscript:

- A rebuttal letter that responds to each point raised by the editor and reviewer(s). You should upload this letter as a separate file labeled 'Response to Reviewers'.
- A marked-up copy of your manuscript that highlights changes made to the original version. You should upload this as a separate file labeled 'Revised Manuscript with Track Changes'.
- An unmarked version of your revised paper without tracked changes. You should upload this as a separate file labeled 'Manuscript'.

Guidelines for resubmitting your figure files are available below the reviewer comments at the end of this letter.

We look forward to receiving your revised manuscript.

Kind regards,

Megan Coffee, MD, PhD
Academic Editor
PLOS Global Public Health

Journal Requirements:

Please review your reference list to ensure that it is complete and correct. If you have cited papers that have been retracted, please include the rationale for doing so in the manuscript text, or remove these references and replace them with relevant current references. Any changes to the reference list should be mentioned in the rebuttal letter that accompanies your revised manuscript. If you need to cite a retracted article, indicate the article’s retracted status in the References list and also include a citation and full reference for the retraction notice.

Additional Editor Comments (if provided):


[Note: HTML markup is below. Please do not edit.]

Reviewers' comments:

Reviewer's Responses to Questions

**Comments to the Author**

1. If the authors have adequately addressed your comments raised in a previous round of review and you feel that this manuscript is now acceptable for publication, you may indicate that here to bypass the “Comments to the Author” section, enter your conflict of interest statement in the “Confidential to Editor” section, and submit your "Accept" recommendation.

Reviewer #1: All comments have been addressed

Reviewer #2: All comments have been addressed

Reviewer #4: (No Response)

Reviewer #5: (No Response)

2. Does this manuscript meet PLOS Global Public Health’s [publication criteria](https://journals.plos.org/globalpublichealth/s/criteria-for-publication)? Is the manuscript technically sound, and do the data support the conclusions? The manuscript must describe methodologically and ethically rigorous research with conclusions that are appropriately drawn based on the data presented.

Reviewer #1: Yes

Reviewer #2: Yes

Reviewer #4: Yes

Reviewer #5: Yes

3. Has the statistical analysis been performed appropriately and rigorously?

Reviewer #1: Yes

Reviewer #2: Yes

Reviewer #4: Yes

Reviewer #5: Yes

4. Have the authors made all data underlying the findings in their manuscript fully available (please refer to the Data Availability Statement at the start of the manuscript PDF file)?

The [PLOS Data policy](https://journals.plos.org/globalpublichealth/s/data-availability) requires authors to make all data underlying the findings described in their manuscript fully available without restriction, with rare exception. The data should be provided as part of the manuscript or its supporting information, or deposited to a public repository. For example, in addition to summary statistics, the data points behind means, medians and variance measures should be available. If there are restrictions on publicly sharing data—e.g. participant privacy or use of data from a third party—those must be specified.

Reviewer #1: No

Reviewer #2: Yes

Reviewer #4: Yes

Reviewer #5: Yes

5. Is the manuscript presented in an intelligible fashion and written in standard English?

PLOS Global Public Health does not copyedit accepted manuscripts, so the language in submitted articles must be clear, correct, and unambiguous. Any typographical or grammatical errors should be corrected at revision, so please note any specific errors here.

Reviewer #1: Yes

Reviewer #2: Yes

Reviewer #4: Yes

Reviewer #5: Yes

6. Review Comments to the Author

Please use the space provided to explain your answers to the questions above. You may also include additional comments for the author, including concerns about dual publication, research ethics, or publication ethics. (Please upload your review as an attachment if it exceeds 20,000 characters)

Reviewer #1: All of my comments have been addressed

Reviewer #2: (No Response)

Reviewer #4: Overall the authors report on the results of a small pilot RCT to assess the feasibility and acceptability of weekly vs daily vs standard of care SMS reminder with real-time adherence monitoring plus social support using an implementation science informed approach complete with relevant framework/theory. While these are important considerations, the study is hampered by the small sample size and the concurrent strategy components being evaluated (timing, type of message, social support, etc). While the study shows some interesting trends it is a bit hampered by the small sample size.

**Response:** Thank you for this comment. We have now included the following sentence in the study limitation section on page 26.

*Being a pilot study, the small sample size involved limits results generalizability*.

1. In terms of implementation outcomes, in addition to acceptability and feasibility, was reach considered. One challenge with DAT is its potential with perpetuating inequity. Especially as the study excluded individuals who were unable to demonstrate mobile/cellphone use/ownership or signal or ability to maintain and charge, these may be associated with sociodemographic characteristics. While the authors share some content analysis related to underlying SDoH,

**Response:** We thank you for this comments. This type of intervention required participants to have some telephone literacy skills. As indicated in the consort diagram (Figure 1), only 6 TB patients were excluded due to inability to use SMS. And only 10 TB patients were excluded for not having telephones. Interventions specifically targeting illiterate populations can be considered in future.

2. Did the study support/provide airtime for participants in receiving messages? If not, why not? Is this a sustainable model?

**Response:** Thank you for this comment. Receiving SMS in Uganda is free of charge.

3. How was the threshold for feasibility of SMS delivery determined. 66% fidelity to SMS daily while in theory is a “majority,” operationally does not seem reliable enough to consider it feasible.

**Response**: We would like to clarify that as indicated in Table 2, 1913/2395 (80%) of daily SMS were successfully delivered.

4. In terms of the Data Availability Statement, I am unclear about whether requests through the origin IRB constitutes “absence of restriction” as denoted by the Journal’s policy.

**Response:** The data availability statement was derived per the Journal’s guidelines. It clearly states that this is sensitive data but de-identified data can be made available upon request and we also provide details of the IRB committee that will approve the request. Therefore the data will be de-identified upon approval of the request.

5. Finally, some of the statements regarding the results of the univariate and multivariate analyses seem a bit out of proportion to the statistical tests reported. It would be important to note that “trends” of higher adherence are observed, but are not necessarily statistically significant based on the p values described. I encourage the authors to caveat their findings a bit more, especially given the small sample size.

**Response:** We appreciate this comments. We now add the following statement in the section that describes the univariate and multivariate analyses:

*In both the univariate and multivariate analyses, the higher adherence observed was not statistically significant as indicated by the P values.*

6. In the discussion (paragraph 2), many suggestions are made for additional implementation strategy components that more directly address underlying social and structural determinants as barriers for TB affected individuals. However, the logic around increasing “feasibility” of the SMS to social supporters by providing these additional targeted components (financial resources, livelihood support, etc) is unclear. Instead of positioning these novel strategies as a way to increase the feasibility of the SMS, it seems more appropriate to state that these would increase the primary outcome- adherence, and to acknowledge that they may do that with or without the SMS intervention.

**Response**: We appreciate this comment. We have now clarified by re-writing the sentence as follows:

*Complementing the SMS notification intervention with interventions to boost the economic status (**40**) of social supporters (e.g. through income generating activities such as farming) may improve their capacity to provide the support needed by the patients.*

7. In conjunction with comment above, it also might be more appropriate to state that the SMS intervention is not designed to target social and structural determinants of health and so a future consideration would be linking this strategy to one that does in order to maximize not just feasibility but overall effect.

**Response:** Thank for this comments. We agree with this suggestion, which is well incorporated in the response for the above comment.

8. Paragraph starting line 415 requires references.

**Response:** Thank you for this comment. The paragraph was intended to emphasize the findings from this study. This has now been made clear as shown below.

***“****Overall, our findings indicate that real-time adherence monitoring linked to SMS reminders to the PLTB and notifications to social supporters is feasible and acceptable for supporting TB drug administration.****”***

9. Discussion paragraph starting at line 424 could be improved by differentiating the reviews/prior literature on real-time adherence monitoring linked to SMS vs other SMS reminder systems.

**Response:** We have refined the paragraph to differentiate the two interventions.

Reviewer #5: The authors report on the results of a pilot randomized controlled trial among patients initiating treatment for drug-sensitive TB in a low-resource setting with high HIV co-infection in Uganda. It was a three arm ‘pilot’ trial, based on prior informative studies, whereby all participants received real-time electronic adherence monitors and were randomized (1:1:1) to daily or weekly SMS reminders to both patients and their identified social supporters for the first 3 months followed by only triggered SMS sent when the pill bottle was not opened for the remainder of the treatment course, vs a control arm that did not use SMS but dose monitor bottles only. They assessed acceptability, feasibility, and percent adherence levels by the real-time monitors as a primary outcome. As a 'pilot' RCT, it was not powered to detect a meaningful clinical outcome. Overall, this appears to be a well conducted study and is well presented. The inclusion of text messages to social supporters is interesting and appears to be an advancement over previous versions these authors have studied in terms of integrated adherence support linked to the monitoring. This use of the dose monitor plus texting approach in TB and including social supporters in receiving SMS messages is novel and should be of interest to people running TB programs and looking for alternative to in-person DOT. There are no major weaknesses I am concerned about, but a few minor suggestions.

Minor

Title/Abstract:
The title and/or abstract should include the study location/country for better context.

**Response:** We thank the reviewer for this comment, we have now revised the title to read as below

***The Feasibility, Acceptability, and Preliminary Impact of Real-Time Monitors and SMS on Tuberculosis Medication Adherence in Southwestern Uganda: Findings from a Mixed Methods Pilot Randomized Controlled Trial***

People/persons – suggest choose one. Also, PLTB often stands for Pleural TB in the existing literature, if not a standard acronym, perhaps avoid it or select a unique one.

**Response:** We have used TB patients instead.

One concern is that if SMS messages are used as ‘reminders’ for daily dosing, then 80% ‘sent’ rate would not be considered adequately feasible, as 20+ % lack of reminders may negatively influence adherence rates if they are in fact relied upon as reminders. If >80% of adherence is an expected minimum level for clinically effective treatment, this already starts at the bottom end? Also, if 96% of the texts were sent to social reports about adherence data, but this is more than the success rate to the patients, its hard to understand how the not sent messages to patients would be reported to social supporters? That said, the higher adherence levels in the daily SMS vs other arms is reassuring. Perhaps this can be discussed.

**Response:** We appreciate this comment. We believe that overall, 80% SMS reminder ‘sent’ rate is reasonably feasible, as the feasibility metric for most technological interventions is often at 70%.

Also, we would to clarify that SMS reminders and notifications were automatically sent to patients and social supporters respectively and concurrently. Likewise, the reports about SMS reminders and notifications sent were system generated. So, the system would still send SMS notifications to social supporters regardless of whether or not the SMS reminders were successfully sent to patients.

If all PLTB as stated found the intervention useful, then why and what additional features need to be included to overcome concerns of TB status disclosure and effects of poverty? I’m not sure this is a data derived statement.

**Response:** We thank the reviewer for this comment. We would like to clarify that although all participants reported (through self-reported linkert-scaled surveys) the intervention to be useful, in-depth data collected from qualitative interviews discovered some challenges of TB status disclosure and structural barriers to medication adherence as indicated in Table 4.

Line 60 – Clarify “Only real-time monitoring creates the potential for intervening prior to the development of treatment failure and/or drug resistance” Clearly there are other ways to assess adherence and how the patients are doing prior to treatment failure or drug resistance.

**Response:** We thank you for this comment. We have now refined the statement to read:

*“Real-time monitoring creates the potential for intervening prior to the development of treatment failure and/or drug resistance.”*

Introduction:
Overall, it is well written and justified.
Line 82: Please provide a level and reference for the key statement “treatment adherences remain a challenge”, since this is core to the purpose of the study.

**Response:** We thank you for this comment. The reference has now been added

Study Design and Setting are fairly well described, but please include more details on standard of care adherence support, since this is critical context to the additional or interactive effects of the study interventions. For instance, what adherence training or support is given upfront. And if patients are seen in clinic/person every 2 weeks, what is the purpose of the weekly ‘reminder’ (not dosing which is daily, and not appointments, which are biweekly?).

**Response:** We appreciate this comment. In this same section, we indicated that TB patients self-administer the TB therapy. We have now added this sentence:

“The clinic verbally encourages patients to adhere to their medication”.

The purpose of weekly reminders was to remind them take their medication. Although the medication is taken daily, receiving weekly reminders can generally encourage patients to always take medication on time. Although it was not reported by participants in this study, some patients in the SMS-based medication adherence previous studies reported the possibility of fulling up their inboxes with daily messages. So, the pilot study reported in this manuscript explored both daily and weekly to assess patient preferences in order to inform the second phase of the study which is on-going.

Inclusion criteria – includes only people that can text (see below re estimating cellphone access and ability). If purposive sampling was used, would the assessed/screened represent the whole clinic population or was there screening bias? This is important in estimating accessibility for the intervention in the whole clinic context.

**Response:** Thank you for this comment. As indicated, the participants included in the study were purposively selected based on the knowledge of the intervention since participants needed to read and understand the SMS. As indicated above, this type of intervention required participants to have some telephone literacy skills. As indicated in the consort diagram (Figure 1), only 6 TB patients were excluded due to inability to use SMS. And only 10 TB patients were excluded for not having telephones. Interventions specifically targeting illiterate populations can be considered in future.

Line 153: please indicate which way purposive sampling was used to skew gender from clinic norms, and any other factors that may have influence recruitment bias. Consecutive would provide better whole populations estimates for factors related to inclusion criteria.

**Response:** As indicated in the section for study procedures (page 8), a random number generator (random allocation was generated by the study coordinator) was used to determine study arm assignments by the study team. After screening and consenting, all TB participants received a real-time adherence monitoring device and were randomized 1:1:1. The sentence about sampling by gender was from the qualitative formative study which was carried out initially—this sentence does not belong to this manuscript and has thus been deleted.

The Wisepill technology is fairly well described. Perhaps discussion of if separate SIM cards are required or if they are linked to patients phones, and which networks can be included, would be additionally helpful.

**Response:** Thank you for this comment. The Wisepill devices have an internal modem and subscriber identity module (SIM) card enable the device to send a real-time mobile signal to a secure Web server (hosted in South Africa) by General Packet Radio Service. Receipt of this signal was taken as a proxy for taking medication. The SIM card is embedded within the device, and is not linked to the patients’ phones. Wisepill server has an interface for inputting patient’s phone numbers to enable the reception of SMS reminders. The following statement has been added on pages 7&8.

*The real-time adherence monitor has an internal modem and subscriber identity module (SIM) card enable the device to send a real-time mobile signal to a secure web server (hosted in South Africa) by General Packet Radio Service.*

Line 198: Please include more information on who the social supporters were (e.g. inclusion/exclusion criteria). If they were required for all participants (and how many might not have met this criteria) and what their roles were given that they were ‘not given specific instructions’ (authors only state they explained expectations, but no what those were). See disclosure issue below as well.

**Response:** We thank you so much for this information. The eligibility criteria for social supporters has been clearly documented under the selection of study participants section on page 7 as show below:

*Inclusion criteria for social supporters were a) being age 18 years and older, b) living in the Mbarara District, c) having an ongoing relationship with the people living with TB (e.g., friend or family member), d) owning a cell phone for personal use, and e) knowing how to use SMS. The only exclusion criterion was being unwilling or unable to give consent.*

Also, we have now revised the sentence about the role of social supporters to read:

Social supporters were generally encouraged to provide support (e.g. medication reminders, counseling, or transport to the clinic) necessary for enabling study participants take their medication well, if possible.

Line 231: Loss to follow-up not included, therefore per-protocol study (vs ITT). Line 236: ITT secondary analysis (it might be clearer to describe PP vs ITT upfront as its mentioned in the discussion)

**Response:** We appreciate this comment. This has now been revised (page 11) as shown below:

*Adherence data for people living with TB who died was included up until death; data were also excluded at loss to follow-up (at the onset of missing data), which was defined as having no contact or adherence data for two months per protocol analysis.*

Line 281: What is meant by social supporters’ co-infection with HIV?

**Response:** We apologize for this confusion. This has been now revised to mean that 16 (38%) of social supporters had HIV as shown below:

*Of the 42 social supporters, 28 (66%) were female, 16 (38%) had HIV, and 32 (22%) had no regular income.* on Page 13.

Table 1: Given the question was asked of PLTB if they had ‘enough social support’, it would be interesting given the nature of the intervention to describe the experiences of the subgroup who initially reported ‘not enough social support’ at baseline and if there was any finding worth exploring in future innovations or studies.

**Response:** We agree with the reviewer about the suggested approach. Unfortunately, we did not follow-up this subgroup to explore their experiences at end-line.

Disclosed TB status – please explain how can 14-20% not have disclosed to anyone other than a healthcare provider be but all identified social supporters to receive texts?

**Response:** Thank you for this comment. To clarify, the inclusion criteria for social supporters were a) being age 18 years and older, b) living in the Mbarara District, c) having an ongoing relationship with the TB patients (e.g., friend or family member), d) owning a cell phone for personal use, and e) knowing how to use SMS. Since having disclosure TB status to social supporters was not an inclusion criterion, it is possible that some patients provided social supporters whom they had not yet disclosed to, but were comfortable disclosing to.

Line 291: All found SMS easy to use. Is this selection bias? What is the baseline literacy and texting rate in the clinic population?

**Response**: In the section for selection of study participants (page 7), we indicate that *knowing how to use SMS* was among the selection criteria for TB patients. It is therefore not surprising that all patients found the study-related SMS easy to use.

Line 295: States SMS reminders occurred when the electronic adherence monitor was opened. But isn’t the point of a reminder to occur before medication is taken? Sentence may need clarification.

**Response:** We thank you for highlighting this. It has now been clarified as below on page 15

*The sending of these SMS reminders occurred when the electronic adherence monitor had already been opened by the patients to take medication, but there was delay or absence of the corresponding signal due technical issues (such as poor network coverage).*

Line 298: Yes, since the SMS text are medication ‘reminders’ one would expect daily reminders timed with dosing schedules. This was confirmed by patients in line 302. Note, HIV studies that use weekly texts were not reminders, they were checkins, or informational messages. The sentence in line 298 has a wording issue stating “SMS reminders to triggered SMS reminders” What does this mean?

**Response:** We thank you for this comment. As described above, in this study, the purpose of weekly reminders was to remind them take their medication. Although the medication is taken daily, receiving weekly reminders can generally encourage patients to always take medication on time. Although it was not reported by participants in this study, some patients in the SMS-based medication adherence previous studies reported the possibility of fulling up their inboxes with daily messages. So, the pilot study reported in this manuscript explored both daily and weekly to assess patient preferences in order to inform the second phase of the study which is on-going.

We apologize for this confusion. The wording issue in line 298 has now been clarified as below:

*Most TB patients in the weekly SMS arm (n=17; 89%) wished they had received daily SMS reminders to triggered SMS reminders.*

Interesting finding about preferences of personalized messages to default ‘reminders’.
Table 2. As above, if only 80% of medication reminders are sent/received as expected, this means they would not be feasible/reliable as ‘dose reminders’ as this is below desired adherence levels? (albeit they may have other general benefits?)

**Response:** As described above, we believe that overall, 80% SMS reminder ‘sent’ rate is reasonably feasible, as the feasibility metric for most technological interventions is often at 70%.

SMS notifications and social supporters – this is the most novel and fascinating part of the study and is well described. The financial and social support delineation is very interesting.
Real-time adherence monitor – patient acceptability and ease of use is encouraging. However, data on the clinic population as a whole would be important as inclusion criteria may have excluded less literate and technically capable patients.

**Response:** We agree with the reviewer regarding the non-inclusion of the less literate; As described in our inclusion criteria, this study focused on patients who can use SMS.

Table 3 is well presented and contains useful technical and contextual information for the readers.

**Response:** Thank you for appreciating

Discussion of risks:
Since patients reported usefulness of the SMS to remind them to take their daily medications, discuss the risk of the 20% delayed or not delivered reminders. Are there alternatives, or does the data indicated it doesn’t matter as good habits are formed?

**Response:** Thank you for this comment. Since SMS were sent to patients and to their social supporters as notifications, there was a possibility of patients being reminded to take their medication by their social supporters after receiving the notifications. This could have potentially covered up for the 20% delayed or undelivered SMS reminders to patients.

Table 4 has excellent illustrative examples of quotations for context.
Primary adherence data is well presented.
**Discussion**
The discussion is balanced and reflects the key findings. Perhaps accessibility/acceptability is overstated if intended to reflect the whole PLTB clinic population. What about the others, how would they be treated or supported in such a program?

**Response:** As indicated in the limitation section, b*eing a pilot study, the small sample size involved limits results generalizability*.

A larger trial is recommended. Which features would the authors recommend? It seems to me the weekly ‘reminders’ arm could be discontinued due to lower effect and patient preference compared to daily. This would be a reasonable conclusion to include.

**Response:** We totally agree with the reviewer about dropping weekly SMS in a larger study.

The involvement and experience of social supporters via SMS is novel and interesting.
Line 431 states this technology can keep PLTB connected with healthcare workers, but it wasn’t clear from this study how that was the case (vs social supporters).

**Response:** We appreciate this comment. We have now revised the sentence to read:

This technology can keep TB patients connected with healthcare supporters, which can potentially minimize the challenge of social isolation that is often associated with TB.

7. PLOS authors have the option to publish the peer review history of their article ([what does this mean?](https://journals.plos.org/globalpublichealth/s/editorial-and-peer-review-process#loc-peer-review-history)). If published, this will include your full peer review and any attached files.


**Do you want your identity to be public for this peer review?** If you choose “no”, your identity will remain anonymous but your review may still be made public.


For information about this choice, including consent withdrawal, please see our [Privacy Policy](https://www.plos.org/privacy-policy).

Reviewer #1: No

Reviewer #2: **Yes:** Haron Gichuhi

Reviewer #4: No

Reviewer #5: No

[NOTE: If reviewer comments were submitted as an attachment file, they will be attached to this email and accessible via the submission site. Please log into your account, locate the manuscript record, and check for the action link "View Attachments". If this link does not appear, there are no attachment files.]

While revising your submission, please upload your figure files to the Preflight Analysis and Conversion Engine (PACE) digital diagnostic tool, <https://pacev2.apexcovantage.com/>. PACE helps ensure that figures meet PLOS requirements. To use PACE, you must first register as a user. Registration is free. Then, login and navigate to the UPLOAD tab, where you will find detailed instructions on how to use the tool. If you encounter any issues or have any questions when using PACE, please email PLOS at [figures@plos.org](mailto:figures@plos.org). Please note that Supporting Information files do not need this step.
